# Nanoparticle Targeting in Chemo‐Resistant Ovarian Cancer Reveals Dual Axis of Therapeutic Vulnerability Involving Cholesterol Uptake and Cell Redox Balance

**DOI:** 10.1002/advs.202305212

**Published:** 2024-01-23

**Authors:** Yinu Wang, Andrea E. Calvert, Horacio Cardenas, Jonathon S. Rink, Dominik Nahotko, Wenan Qiang, C. Estelle Ndukwe, Fukai Chen, Russell Keathley, Yaqi Zhang, Ji‐Xin Cheng, C. Shad Thaxton, Daniela Matei

**Affiliations:** ^1^ Department of Obstetrics and Gynecology Feinberg School of Medicine Northwestern University Chicago IL 60611 USA; ^2^ Simpson Querrey Institute for BioNanotechnology Feinberg School of Medicine Northwestern University Chicago IL 60611 USA; ^3^ Division of Hematology/ Oncology Department of Medicine Feinberg School of Medicine Northwestern University Chicago IL 60611 USA; ^4^ Center for Developmental Therapeutics,Feinberg School of Medicine Northwestern University Evanston IL 60208 USA; ^5^ Robert H. Lurie Comprehensive Cancer Center Northwestern University Chicago IL 60611 USA; ^6^ Department of Physics Boston University Boston MA 02215 USA; ^7^ Department of Urology Feinberg School of Medicine Northwestern University Chicago IL 60611 USA; ^8^ Jesse Brown Veteran Affairs Medical Center Chicago IL 60612 USA

**Keywords:** cholesterol uptake, ferroptosis, platinum resistant ovarian cancer, redox targeting

## Abstract

Platinum (Pt)‐based chemotherapy is the main treatment for ovarian cancer (OC); however, most patients develop Pt resistance (Pt‐R). This work shows that Pt‐R OC cells increase intracellular cholesterol through uptake via the HDL receptor, scavenger receptor type B‐1 (SR‐B1). SR‐B1 blockade using synthetic cholesterol‐poor HDL‐like nanoparticles (HDL NPs) diminished cholesterol uptake leading to cell death and inhibition of tumor growth. Reduced cholesterol accumulation in cancer cells induces lipid oxidative stress through the reduction of glutathione peroxidase 4 (GPx4) leading to ferroptosis. In turn, GPx4 depletion induces decreased cholesterol uptake through SR‐B1 and re‐sensitizes OC cells to Pt. Mechanistically, GPx4 knockdown causes lower expression of the histone acetyltransferase EP300, leading to reduced deposition of histone H3 lysine 27 acetylation (H3K27Ac) on the sterol regulatory element binding transcription factor 2 (SREBF2) promoter and suppressing expression of this key transcription factor involved in the regulation of cholesterol metabolism. SREBF2 downregulation leads to decreased SR‐B1 expression and diminished cholesterol uptake. Thus, chemoresistance and cancer cell survival under high ROS burden obligates high GPx4 and SR‐B1 expression through SREBF2. Targeting SR‐B1 to modulate cholesterol uptake inhibits this axis and causes ferroptosis in vitro and in vivo in Pt‐R OC.

## Graphical abstract

Platinum resistant (Pt‐R) ovarian cancer (OC) cells increase intracellular cholesterol through uptake via the high‐density lipoproteins (HDL) receptor, scavenger receptor type B‐1 (SR‐B1). SR‐B1 blockade using HDL nanoparticles (NPs) diminishes cholesterol uptake and reduces GPx4 expression leading to Pt‐R OC cell death through ferroptosis, which represents a first‐in‐class approach applicable to Pt‐R OC.

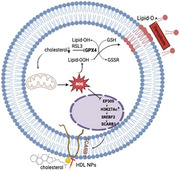

## 1 Introduction

Emergence of platinum (Pt) resistance (Pt‐R) predicts invariably fatal outcomes in high grade serous ovarian cancer (HGSOC) with an expected median survival of 12–18 months.^[^ ^1^, ^2^ ^]^ Pt‐R HGSOC remains an area of unmet need, where progress has remained slow and clinical outcomes are lagging.^[^ ^3^, ^4^, ^5^, ^6^ ^]^ Understanding key vulnerabilities of Pt‐R OC could unlock new possibilities to target HGSOC and improve survival.

While previous studies on Pt‐R have focused on DNA repair pathways or altered membrane transporters, new concepts support metabolic reprogramming as a key contributor to the cellular chemoresistant state. The first step in this transition is caused by a Pt‐induced shift in central carbon metabolism leading to increased oxidative stress.^[^ ^7^ ^]^ Our recent work showed that OC cells become Pt‐R by increasing glutathione (GSH) anti‐oxidative defenses to evade Pt‐induced apoptosis.^[^ ^8^ ^]^ Depletion of GSH‐mediated anti‐oxidant defense mechanisms results in cell membrane phospholipid peroxidation and cell death through a mechanism consistent with ferroptosis, an iron‐dependent form of cell death that results from oxidation of cell membrane polyunsaturated fatty acid (PUFA) tails of cell membrane phospholipids (PUFA‐PL).^[^ ^8^ ^]^ Using single‐cell metabolic imaging based on Raman spectroscopy and isogenic cell lines sensitive or resistant to Pt, we also detected increased lipid uptake and oxidation in Pt‐R cells and tumors.^[^ ^9^ ^]^ Pt‐R OC models were dependent upon fatty acids for energy generation and blockade of fatty acid beta‐oxidation reversed resistance to Pt in vitro and in vivo. Others reported an increased reliance on lipids in HGSOC models.^[^ ^10^ ^]^

Cholesterol is a significant component of cell membranes that modulates fluidity and lipid raft microdomains implicated in oncogenic cellular signaling.^[^ ^11^, ^12^ ^]^ Emerging evidence supports the role of cholesterol in cancer progression and the development of drug resistance^[^ ^13^, ^14^ ^]^ leading to interest in manipulating cholesterol levels to induce anti‐tumor activity. Cells can either synthesize cholesterol de novo in the endoplasmic reticulum (ER) or uptake cholesterol from circulating lipoprotein particles, like high‐ and low‐density lipoproteins (HDL and LDL).^[^ ^12^ ^]^ One strategy to attempt to reduce cell cholesterol is by inhibiting de novo cholesterol biosynthesis using “statins” which block the rate limiting enzyme 3‐hydroxy‐3‐methylglutaryl‐coenzyme A reductase (HMGCR) that converts 3‐hydroxy‐3‐methylglutaryl‐CoA (HMG‐CoA) to mevalonate and, eventually, cholesterol. Interestingly, the use of statins was associated with a reduced risk of developing OC in a case control study^[^ ^15^ ^]^ and with improved survival among women with newly diagnosed OC.^[^ ^16^ ^]^ However, clinical studies reported that addition of statins to standard chemotherapy did not improve clinical outcomes.^[^ ^17^ ^]^ These clinical results are consistent with preclinical findings demonstrating increased uptake, but suppressed de novo biosynthesis, of cholesterol in Pt‐R OC cells,^[^ ^14^ ^]^ supporting a potential strategy targeting OC cell cholesterol uptake.

Cellular cholesterol uptake is mediated by cholesterol‐rich HDL and LDL^[^ ^18^ ^]^ and both lipoproteins types have been implicated in cancer.^[^ ^19^ ^]^ LDLs target the LDL receptor (LDLR) for holoparticle internalization via clathrin mediated endocytosis.^[^ ^20^ ^]^ Cholesterol from the degraded LDL particle is ultimately re‐esterified and stored in cytoplasmic lipid droplets (LDs) or transferred to the cell or mitochondrial membranes via the ER. On the other hand, cholesterol rich HDLs (crHDL) target scavenger receptor type B‐1 (SR‐B1) localized to the cell membrane which transports cholesterol, phospholipids, and other small molecules between the cell membrane and the HDL particle^[^ ^21^ ^]^ and selectively delivers cholesteryl esters to the cell without holoparticle internalization.^[^ ^22^ ^]^ After delivering the core payload of cholesteryl ester, the remnant HDL particle dissociates from SR‐B1. High expression of SR‐B1 has been described as a mechanism of internalizing cholesterol in OC.^[^ ^23^, ^24^ ^]^

Because increased lipid metabolism and cholesterol uptake through SR‐B1 are associated with a Pt and oxidation resistant phenotype in OC, we studied a strategy using synthetic HDL nanoparticles (NP) that target SR‐B1^[^ ^25^, ^26^, ^27^ ^]^ and functionally support the efflux of free cholesterol while blocking the delivery of cholesteryl esters^[^ ^28^, ^29^, ^30^ ^]^ to resistant OC cells and tumors. The functional properties of HDL NP are enabled by employing an inert nanoparticle “core” as a template to assemble phospholipids and protein (i.e., apolipoprotein A‐I) in a manner that approximates the surface chemistry of native crHDLs that bind SR‐B1. Different from native crHDL, HDL NPs are synthesized without cholesterol in the outer layer of phospholipids, which enables maximal free cholesterol efflux from the cell membrane and HDL NPs have no internal cholesteryl ester cargo to deliver to the target cell.^[^ ^27^, ^31^, ^32^, ^33^ ^]^ Compositional differences between the HDL NPs and crHDLs functionally differentiate the two materials. Here we report that Pt‐R OC cells and tumors are rich in intracellular cholesterol and HDL NP targets SR‐B1 to deplete cholesterol stores in Pt‐R OC cells. We found that GPx4 expression, highly upregulated in Pt‐R cells, is intimately linked with cell cholesterol stores and SR‐B1 expression. Collectively, targeting SR‐B1, cell cholesterol and GPx4 is a unique mechanism to induce cell death through ferroptosis and block Pt‐R ovarian tumor growth.

## 2 Results

### 2.1 Pt‐R OC Cells Demonstrate Increased Intracellular Cholesterol Accumulation

**Figure 1:**
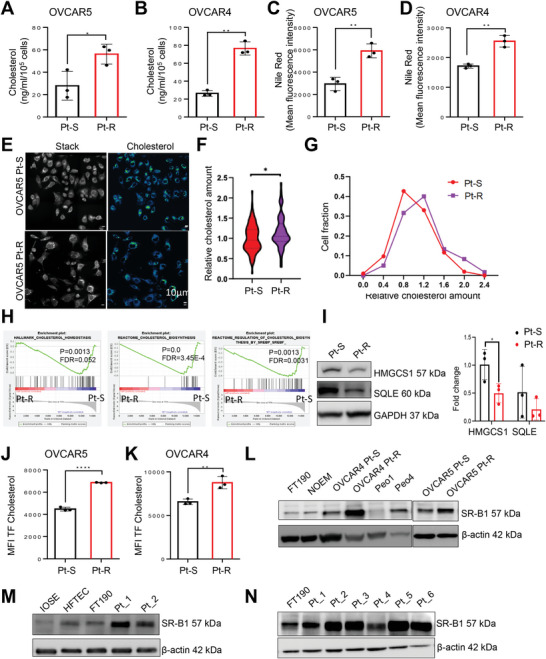
Platinum resistant OC cells are more dependent on cholesterol import than on de novo synthesis for survival. A,B) Intracellular total cholesterol level (mean ± SD, *n* = 3) measured using an Amplex Red cholesterol assay kit in Pt‐S and Pt‐R variants of OVCAR5 (A) and OVCAR4 (B) OC cells. C,D) Total lipid levels in OVCAR5 (C) and OVCAR4 (D) Pt‐S and Pt‐R cells were measured by Nile Red staining and analyzed by flow cytometry. Values represent median fluorescent intensity (MFI; mean ± SD, *n* = 3). E) Representative stimulated Raman scattering (SRS) images (left panel) and unmixed cholesterol channel signal images (right panel) of OVCAR5 Pt‐S and Pt‐R OC cells. F) Quantification of the SRS cholesterol channel signal intensity from OVCAR5 Pt‐S and Pt‐R OC cells relative to total cholesterol levels and G) at cell fraction level. H) GSEA enrichment plots of gene sets involved in de novo cholesterol synthesis: “hallmark cholesterol homeostasis,” “reactome cholesterol biosynthesis,” and “reactome regulation of cholesterol biosynthesis by SREBP/F” in OVCAR5 Pt‐S versus Pt‐R cells measured by RNA‐seq (*n* = 3). I) (Left) Western blot images of enzymes HMGCS1 and SQLE regulating de novo cholesterol biosynthesis in OVCAR5 Pt‐S and Pt‐R cells. Quantification of band intensity was performed by Image J and is shown in the right panel (*n* = 3). J,K) Cholesterol uptake in OVCAR5 (J) and OVCAR4 (K) Pt‐S and Pt‐R cells measured using TopFluor Cholesterol by flow cytometry. Values represent median fluorescent intensity (MFI; mean ± SD, *n* = 3). L) Western blot of SR‐B1 protein in immortalized fallopian tube epithelial cells (FT‐190), normal endometrial cells (NoEM), OVCAR5, and OVCAR4 Pt‐S and Pt‐R cells, and in the isogenic Pt‐S Peo1 and Pt‐R Peo4 OC cells (*n* = 3). M) SR‐B1 protein expression levels in immortalized ovarian surface epithelial cells (IOSE), normal human fallopian tube epithelial cells (HFTEC), FT190, and two primary cells derived from HGSOC tumors (*n* = 2). N) SR‐B1 protein levels measured by western blot in OC tumors (*n* = 6) and in FT190 cells. For all panels, *p < 0.05, **p < 0.01, ***p < 0.001, ****p < 0.0001.

We developed Pt‐R OC models through repeated exposure of OC cell lines to cytotoxic doses of Pt.^[^ ^8^, ^34^ ^]^ Compared to platinum‐sensitive (Pt‐S) cells, the Pt‐R cells demonstrated increased antioxidant capacity (upregulated GPx4), increased susceptibility to ferroptosis,^[^ ^8^ ^]^ and increased fatty acid (FA) accumulation and import.^[^ ^9^ ^]^ Given the reported dependence of other cancer cells to cholesterol,^[^ ^13^, ^35^, ^36^ ^]^ we measured intracellular total cholesterol levels through an Amplex Red colorimetric assay in OC Pt‐S and Pt‐R cells. Total cholesterol abundance, including free cholesterol and cholesteryl esters, was higher in Pt‐R compared to Pt‐S OVCAR5 and OVCAR4 cells (**Figure** **1**A,B). As cholesterol is a key component of lipid droplets (LDs), we also measured total LD content in OVCAR5 and OVCAR4 Pt‐R and Pt‐S cells by using Nile Red staining and quantification of mean fluorescence intensity (MFI) via flow cytometry. Increased LD content was demonstrated in Pt‐R versus Pt‐S OVCAR5 and OVCAR4 cells (Figure 1C‐D). LDs contain tryglicerides (TAGs) along with cholesterol, therefore, we also measured TAGs content. TAGs were increased in both OVCAR5 and OVCAR4 Pt‐R cells versus Pt‐S cells (Figures S1A,B, Supporting Information). Spectroscopic stimulated Raman scattering (SRS) imaging maps cholesterol content by using its unique signature in the high wave number CH vibration window. SRS imaging of Pt‐S and Pt‐R cancer cell populations quantified cholesterol content and showed increased cholesterol rich cell populations in Pt‐R versus Pt‐S cells (Figure 1E–G). Thus, several quantitative methods demonstrated increased intracellular cholesterol and TAGs in Pt‐R versus Pt‐S OC cells.

Next, we investigated whether increased cholesterol content resulted from de novo biosynthesis or increased uptake. RNA sequencing comparing Pt‐R and Pt‐S OVCAR5 cells (GSE 148 003) demonstrated downregulation of genes related to cholesterol synthesis among differentially expressed genes (DEGs). Gene set enrichment analysis (GSEA) showed significant enrichment of gene sets related to “*hallmark cholesterol homeostasis*” (Figure S1C, Supporting Information), “*reactome cholesterol biosynthesis*” and “*reactome regulation of cholesterol biosynthesis by SREBP/F*” in OVCAR5 Pt‐S versus Pt‐R cells (Figure 1H), suggesting downregulation of cholesterol synthesis gene sets in resistant cells. Genes implicated in these pathways are listed in Tables S1 and S2, Supporting Information. Two important enzymes implicated in de novo cholesterol biosynthesis, 3‐hydroxy‐3‐methylglutaryl Coenzyme A synthase 1 (HMGCS1) and squalene monooxygenase (SQLE), were downregulated in Pt‐R compared with Pt‐S OC cells (Figure 1I). Given the observations supporting downregulation of cholesterol biosynthesis in Pt‐R cells, we next examined cholesterol uptake. The TopFluor‐labeled cholesterol uptake assay demonstrated increased cholesterol uptake in Pt‐R OVCAR5 (Figure 1J) and OVCAR4 (Figure 1K) compared to Pt‐S cells, suggesting that Pt‐R cells are dependent on cholesterol uptake to maintain high intracellular cholesterol levels. Further, the expression levels of the scavenger receptor type B‐1 (SR‐B1), a high‐affinity HDL receptor,^[^ ^37^ ^]^ were increased in Pt‐R OVCAR4 and OVCAR5 compared with Pt‐S cells and in Peo4 (Pt‐R) versus Peo1 (isogenic Pt‐S) cell lines (Figure 1L). SR‐B1 protein expression was higher in a majority of OC cell lines compared to normal endometrial cells (NoEM),^[^ ^38^ ^]^ and immortalized fallopian tube epithelial cells (FT‐190) (Figure 1L). Upregulated SR‐B1 protein expression was also validated in primary cells dissociated from HGSOC tumors (*n* = 6, Table S3, Supporting Information) compared with immortalized ovarian surface epithelium (IOSE), primary human fallopian tube epithelial cells (HFTEC), and FT190 cells (Figure 1M,N). In addition, patients with high tumoral SR‐B1 expression as profiled by publically available Gene Expression Omnibus (GEO) databases and The Cancer Genome Atlas (TCGA) databases had significantly reduced overall survival compared to patients with low SR‐B1 expression (*p* = 0.029; Figure S1D, Supporting Information).

### 2.2 HDL NP Inhibit Pt‐R OC Growth

**Figure 2:**
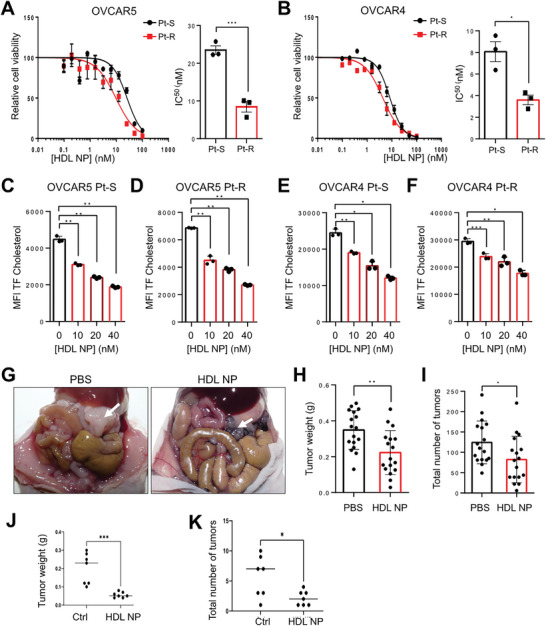
HDL NPs decrease viability of OC cells and inhibit growth of OC tumor xenografts. A,B) Effects of HDL NPs on viability and IC_50_ estimates in Pt‐S and Pt‐R OVCAR5 (A) and OVCAR4 (B) cells treated for 72 (OVCAR4) or 96 h (OVCAR5) and determined using MTS assays. Average IC_50_ (mean ± SD, *n* = 3 replicate experiments) are shown on the right. C–F) Cholesterol uptake (mean ± SD, *n* = 3) measured using TopFluor Cholesterol and flow cytometry in OVCAR5 Pt‐S (C) and Pt‐R (D) and OVCAR4 Pt‐S (E) and Pt‐R (F) OC cells treated with increasing concentrations of HDL NP. G) Representative images of mice injected intraperitoneally with OVCAR5 Pt‐R OC cells and then treated with PBS or HDL NPs. The largest tumors are indicated by arrows. H,I) Total tumor weight (H) and number of tumors (I) in mice treated as described in (G); *n* = 17 per group. J,K). Total tumor weights (J) and numbers of tumors (K) in mice bearing ovarian Pt‐R PDX treated with HDL NPs compared to PBS (*n* = 7 per group). Values are mean ± SD, **p < 0.05, **p < 0.01, ***p < 0.001).

To investigate if SR‐B1‐dependent cholesterol uptake is necessary to sustain OC survival, we employed HDL‐like nanoparticles (HDL NPs) to block SR‐B1. HDL NPs are bioinspired materials that share physicochemical features with native HDLs including size, charge, and surface composition.^[^ ^28^, ^30^, ^32^, ^37^, ^39^ ^]^ OVCAR5 and OVCAR4 Pt‐S and Pt‐R cells were treated with increasing doses of HDL NPs and cell viability was measured. Pt‐R cells were more sensitive to HDL NP treatment compared with Pt‐S cells, as evidenced by lower IC_50_ values (**Figure** **2**A,B, 23.49 ± 1.96 nm (Pt‐S) versus 8.46 ± 2.40 nm (Pt‐R) for OVCAR5 and 8.07 ± 1.60 nm (Pt‐S) versus 3.61 ± 0.76 nm (Pt‐R) for OVCAR4 cells). We hypothesized that the difference in sensitivity was due to a reduction in SR‐B1‐mediated cholesterol uptake and increased dependence of Pt‐R cells on cholesterol. To test this, we examined the uptake of a fluorescently labeledTopFluor (TF) cholesterol in OC cells treated with HDL NP. Treatment with HDL NP reduced cholesterol uptake in both Pt‐S (Figure 2C,E) and Pt‐R (Figure 2D,F) cells.

Treatment with HDL NP also reduced tumor burden in murine models of Pt‐R OC. Pt‐R OVCAR5 cells were orthotopically implanted in the peritoneal cavity of female nude mice. Treatment with PBS (control) or HDL NP was given five times a week (Mon.‐Fri.) i.p. for 4 weeks. HDL NP treatment was well tolerated and did not induce significant changes in body weight (Figure S2A, Supporting Information). Mice treated with HDL NP had decreased tumor burden (Figure 2G), and significantly reduced total tumor weight (Figure 2H) and number of tumors (Figure 2I). Similar results were observed by using a Pt‐R ovarian PDX model. Body weights were not affected by HDL NP treatment (Figure S2B, Supporting Information). PDX tumors disseminated intraperitoneally and displayed HGSOC histological characteristics (Figure S2C,D, Supporting Information). HDL NPs reduced total tumor weight (Figure 2J), number of metastases (Figure 2K), and tumor volume (Figure S2E, Supporting Information). Taken together, these results demonstrate that targeting SR‐B1 by HDL NPs reduces cholesterol uptake and causes decreased cell viability in vitro and tumor growth in vivo.

### 2.3 HDL NPs Decrease the Viability of OC Cells by Inducing Ferroptosis

**Figure 3:**
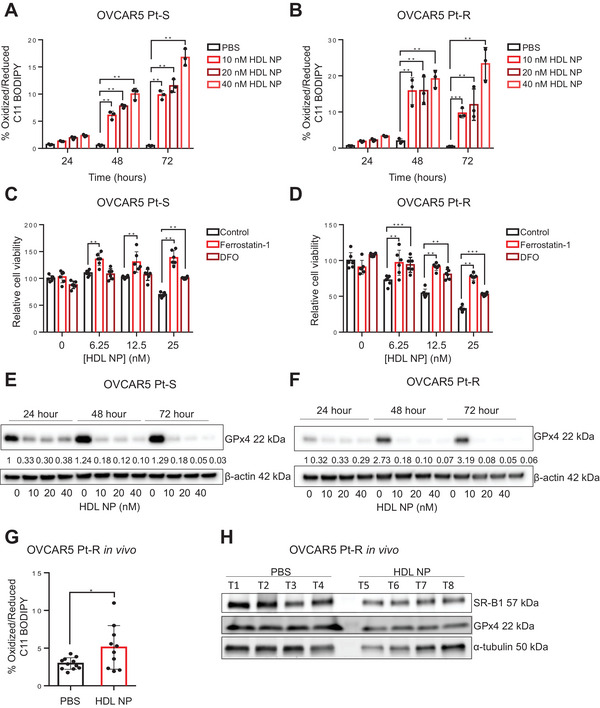
HDL NPs decrease viability of Ovarian Cancer cells by inducing ferroptosis. A,B) OVCAR5 Pt‐S (A) and Pt‐R (B) cells were treated with increasing concentrations of HDL NPs for 24, 48, or 72 h and the percentage of oxidized/reduced lipids was determined by C11 BODIPY staining (mean ± SD, *n* = 3). (C,D) MTS assays measure viability of OVCAR5 Pt‐S (C) and Pt‐R (D) cells treated with increasing concentrations of HDL NPs, alone or in combination with ferrostatin‐1 or DFO. Values are mean ± SD (*n* = 3). E,F) Measurements of GPx4 and β‐actin (loading control) protein levels by western blot in OVCAR5 Pt‐S (E) and Pt‐R (F) cells treated with HDL NPs for 24, 48, or 72 h. Band intensity was quantified using Image J and is indicated below each blot. G) Percentage of oxidized/reduced lipids measured by C11 BODIPY staining in cells isolated from tumor xenografts induced in mice by OVCAR5 Pt‐R cells and treated with PBS or HDL NP (mean ± SD, *n* = 10–11). H) Western blot analysis shows SR‐B1, GPx4, and α‐tubulin (loading control) protein levels in xenografts resected from mice injected i.p. with OVCAR5 Pt‐R cells and treated i.p. with PBS or HDL NPs. Lanes represent tumors from different mice. For all panels, *p < 0.05, **p < 0.0001, ***p < 0.001.

Cells dependent on cholesterol uptake have been shown to be especially sensitive to ferroptosis^[^ ^30^, ^35^, ^40^ ^]^ and we have shown that Pt‐R cells are resistant to pro‐apoptotic signals, but can be killed through ferroptosis.^[^ ^8^ ^]^ We therefore determined whether HDL NP treatment induced increased levels of oxidized lipids in OC cell membranes by using C11 BODIPY staining. HDL NP treatment resulted in a dose‐ and time‐dependent increase in oxidized lipids in both Pt‐S (**Figure** **3**A and Figure S3A, Supporting Information) and Pt‐R cells (Figure 3B and Figure S3B, Supporting Information); however, the magnitude of lipid oxidation was slightly increased in Pt‐R cells. Ferroptosis can be rescued by delivering the membrane‐targeted antioxidant, ferrostatin‐1, or the iron chelator, deferoxamine (DFO). HDL NP‐induced decreased cell viability was, indeed, rescued by ferroptosis inhibitors, ferrostatin‐1 and DFO, (Figure 3C,D and Figure S3C,D, Supporting Information) confirming ferroptosis. Glutathione peroxidase 4 (GPx4) is a key antioxidant enzyme, which reduces oxidized cell membrane polyunsaturated lipids preventing ferroptosis. Western blotting demonstrated that treatment of Pt‐R and Pt‐S OC cells with HDL NPs potently reduced GPx4 protein expression in a dose and time dependent manner (Figure 3E,F and Figure S3E,F, Supporting Information). Furthermore, higher oxidized lipid content was observed in Pt‐R xenografts treated with HDL NP compared to PBS treated tumors (Figure 3G). In addition, Pt‐R tumors from mice treated with HDL NPs had lower GPx4 protein expression than those treated with PBS (Figure 3H). Further, slightly decreased SR‐B1 protein expression levels were noted in HDL NPs treated xenografts (Figure 3H), indicating potential correlation between GPx4 and SR‐B1 expression levels. These data support that HDL NPs induce ferroptosis in Pt‐R OC cells and tumors in vitro and in vivo. Interestingly, the expression levels of HMGCS1, one of the key enzymes regulating de novo cholesterol synthesis, was increased after HDL NPs treatment in OVCAR5 Pt‐S cells (Figure S3G, Supporting Information), but only modestly affected in Pt‐R cells (Figure S3H, Supporting Information), indicating that blocking cholesterol import may induce compensatory activation of cholesterol synthesis. Atorvastatin, a potent inhibitor of cholesterol synthesis, had additive effects on reducing cells viability of Pt‐S OVCAR5 cells in combination with HDL NPs, but did not add cytotoxic effects to HDL NPs in Pt‐R OVCAR5 cells (Figure S3I,J, Supporting Information).

### 2.4 GPx4 Inhibition Re‐Sensitizes OC Cells to Chemotherapy by Targeting SR‐B1 Mediated Cholesterol Import

**Figure 4:**
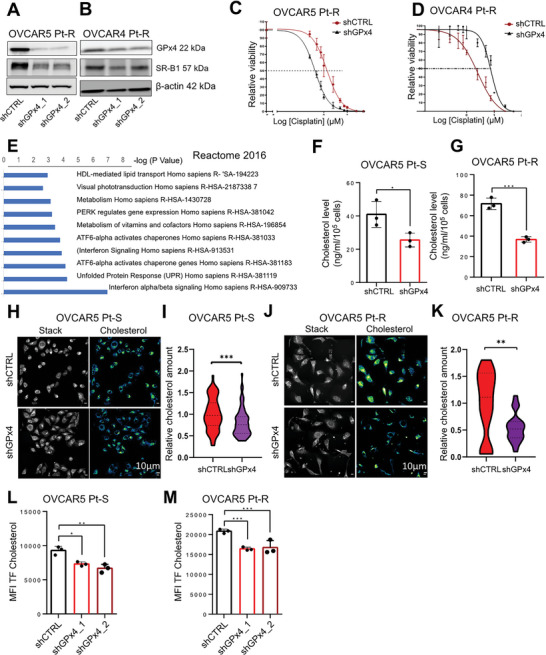
GPx4 inhibition re‐sensitizes OC cells to platinum chemotherapy through reduction of cholesterol uptake. A,B) Western blot shows GPx4, SR‐B1, and β‐actin (loading control) protein levels in OVCAR5 Pt‐R (left panel) and OVCAR4 Pt‐R (right panel) cells transduced with shRNAs targeting *GPx4* (shGPx4) or control shRNAs (shCTRL). shGPx4_1 and shGPx4_2 represent different shRNA sequences. C,D) Cell viability assays measured surviving OVCAR5 (C, *n* = 5 replicates), OVCAR4 (D, *n* = 3 replicates) Pt‐R cells after treatment with cisplatin (various concentrations). E) Reactome analysis of top molecular pathways associated with differentially downregulated genes measured by RNAseq (FDR < 0.05, *n* = 3) in OVCAR5 Pt‐R cells transduced with shRNAs targeting *GPX4* (shGPx4) versus cells transduced with control shRNAs (shCTRL). F,G) Amplex Red cholesterol assay measured total intracellular cholesterol levels/10^5^ cells (mean ± SD, *n* = 3) in shGPx4 versus shCTRL OVCAR5 Pt‐S (F) and Pt‐R (G) OC cells. H–K) Representative SRS images (left) and unmixed cholesterol channel signal images (right) of shCTRL and shGPx4 OVCAR5 Pt‐S (H) and Pt‐R (J) OC cells. Quantification of relative amounts of cholesterol channel signal intensity (mean ± SD, *n* = 3) in shCTRL and shGPx4 OVCAR5 Pt‐S (I) and Pt‐R (K) OC cells. L,M) Cholesterol uptake measured by TopFluor Cholesterol flow cytometry in shGPx4 and shCTRL OVCAR5 Pt‐S (L) and Pt‐R (M) cells (mean ± SD, *n* = 3). For all panels, *p < 0.05, **p < 0.01, ***p < 0.001.

Due to the observed correlation between GPx4 and SR‐B1 expression in Pt‐R cells, we investigated whether SR‐B1‐regulated cholesterol homeostasis in OC cells is linked to the antioxidant enzyme GPx4. We knocked‐down (KD) GPX4 by stable shRNA transfection in Pt‐R OC cells. Decreased GPx4 protein expression was confirmed by western blotting in Pt‐S and Pt‐R OVCAR5 and OVCAR4 cells transduced with two shRNA sequences targeting GPx4 versus control shRNA (shCTRL; **Figure** **4**A,B, Figure S4A,B,E,G, Supporting Information) and response to Pt was measured in cells in which the protein was downregulated. GPx4 KD decreased the IC_50_ to Pt by approximately twofold in Pt‐R OVCAR5 (Figure S4C, Supporting Information) and OVCAR4 cells (Figure S4D, Supporting Information), and by ≈1.5‐fold in Pt‐S OVCAR5 (Figure S4F, Supporting Information) and OVCAR4 cells (Figure S4H, Supporting Information). Similarly, treatment with HDL NPs, which depletes cancer cells of GPx4, sensitized Pt‐R cancer cells to carboplatin. Combination of HDL NPs and carboplatin synergistically killed OVCAR5 Pt‐R cells compared with either treatment alone (combination index (CI), CI = 0.825 at ED_50_, CI = 0.924 at ED_75_, Figure S4I,J, Supporting Information).

To understand whether modulation of GPx4 expression alters pathways related to cholesterol uptake or biosynthesis, an unbiased approach was pursued by using transcriptomic analyses. RNA sequencing of Pt‐R OVCAR5 cells transfected with shGPx4 versus shCTRL revealed 3919 DEGs (FDR<0.05, see GSE234404). “Reactome analysis” of top molecular pathways associated with the downregulated DEGs in Pt‐R OVCAR5 cells transfected shGPx4 versus shCTRL cells included, among the top altered gene sets, HDL‐mediated lipid transport (Figure 4E). In addition, GPx4 KD in OVCAR5 Pt‐S and Pt‐R OC cells reduced SR‐B1 expression (Figure 4A,B, Figure S4E,G, Supporting Information), indicating that GPx4 is linked to cholesterol accumulation in Pt‐R OC cells by modulating SR‐B1 and HDL‐mediated cholesterol uptake. Therefore, we examined the impact of GPx4 KD on cholesterol accumulation on OVCAR5 Pt‐S and Pt‐R cells. An Amplex Red cholesterol assay indicated that total intracellular cholesterol levels were reduced in OVCAR5 Pt‐S (Figure 4F) and Pt‐R cells (Figure 4G) in which GPx4 was knocked down compared with shCTRL transfected cells. SRS imaging analysis also demonstrated decreased intracellular cholesterol signal intensity in OVCAR5 Pt‐S (Figure 4H,I and Figure S4K, Supporting Information) and Pt‐R (Figure 4J,K and Figure S4L, Supporting Information) cells in which GPx4 was KD compared with control cells. To determine whether the effects of GPx4 on cholesterol content were contributed by altered cholesterol uptake, we used the TopFluor cholesterol uptake assay. FACS analysis demonstrated that GPx4 KD reduced cholesterol uptake in OVCAR5 Pt‐S (Figure 4L), and Pt‐R cells compared with control cells (Figure 4M). Collectively these data support that GPx4 expression is linked to intracellular cholesterol homeostasis and cholesterol uptake, which contribute to chemoresistance in OC cells.

### 2.5 GPx4‐Mediated Cholesterol Accumulation Maintains Cellular Redox Homeostasis in OC Cells

**Figure 5:**
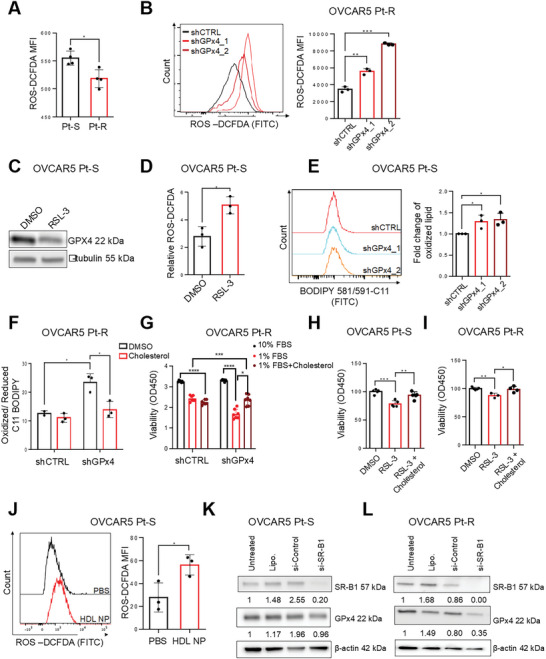
GPx4‐mediated cholesterol accumulation maintains cellular redox homeostasis in OC cells. A) Quantification of intracellular ROS levels in OVCAR5 Pt‐S and Pt‐R OC cells. ROS levels are represented by mean DCFDA fluorescence intensity (± SD, *n* = 3) as quantified with a fluorescence plate reader at Ex/Em = 485/535 nm. B) Histograms (left) and quantification of intracellular ROS levels with DCFDA (right) in OVCAR5 Pt‐R cells transduced with shRNAS directed at GPx4 (shGPx4_1 and shGPx4_2) or control shRNA (shCTRL). Results show mean ± SD (*n* = 3) of DCFDA fluorescence intensity quantified by flow cytometry. C) Western blot measures GPx4 and α‐tubulin (loading control) protein levels in OVCAR5 Pt‐S cells treated with DMSO or the GPx4 inhibitor RSL‐3 (200 nm, 48 h, *n* = 3). D) Intracellular ROS levels in OVCAR5 Pt‐S OC cells treated with RSL‐3 (1 um, 4 h) versus DMSO, detected by DCFDA fluorescence and quantified with a fluorescence plate reader. E) Assessment of intracellular lipid peroxidation using BODIPY 581/591‐C11. Histograms of fluorescence intensities (left) and fold‐change of fluorescence intensities (mean ± SD, *n* = 3) (right) in shGPx4 versus shCTRL OVCAR5 Pt‐S OC cells. F) Effects of cholesterol (50 µg mL^−1^ for 48 h) on percentage of oxidized/reduced lipids measured by flow cytometry of C11 BODIPY in shCTRL and shGPx4 OVCAR5 Pt‐R cells cultured in low serum conditions (1% FBS) (mean ± SD, *n* = 3). G) Viability of OVCAR5 Pt‐R cells, cultured in full serum (10% FBS), basal conditions (1% FBS), and 1% FBS + cholesterol (50 µg mL^−1^). Cell viability was measured with the CCK8 assay at 48 h (mean ± SD, *n* = 3–5 replicates). H,I) Viability of OVCAR5 Pt‐S (H) and Pt‐R (I) OVCAR5 OC cells, cultured in low serum conditions (1% FBS), and treated with DMSO, GPx4 inhibitor RSL‐3 (200 nm) or RSL‐3 plus cholesterol (50 µg mL^−1^) combination. Cell viability was measured with the CCK8 assay at 96 h (Pt‐S) or 48 h (Pt‐R) (mean ± SD, *n* = 3–5). J) Histograms (left) and quantification of intracellular ROS levels (right) by DCFDA fluorescence intensity determined by flow cytometry (mean ± SD, *n* = 3) in OVCAR5 Pt‐S OC cells treated with PBS or HDL NPs (40 nm, 24 h). K,L) Western blot analysis of SR‐B1, GPx4, and β‐actin (loading control) in OVCAR5 Pt‐S (K) and Pt‐R (L) cells transfected with siRNA against SR‐B1. Band intensity was measured with Image J and is indicated below each blot. For all panels, *p < 0.05, **p < 0.01, ***p < 0.001.

We previously reported that Pt‐R OC cells harbor enhanced glutathione metabolism, including increased GPx4,^[^ ^8^ ^]^ which protects cells from chemotherapy‐induced oxidative stress and that reducing intracellular reactive oxygen species (ROS), especially lipid‐orientated ROS (L‐ROS), is critical to regulating chemotherapy resistance.^[^ ^8^, ^41^ ^]^ At the same time, these Pt‐R cells are enriched in cholesterol and dependent upon cholesterol uptake. To determine whether cholesterol homeostasis plays a role in regulating cellular ROS levels, the effects of GPx4 and of cholesterol uptake blockade on ROS levels were quantified. First, intracellular ROS levels were measured through 2',7'‐dichlorofluorescin diacetate (DCFDA) staining and found to be decreased in Pt‐R versus Pt‐S OC cells (**Figure** **5**A, Figure S5A, Supporting Information). Intracellular ROS levels increased if GPx4 was KD (Figure 5B) or inhibited by using the small molecule inhibitor RSL3 (Figure 5C,D). These data support that GPx4 reduces cellular ROS in Pt‐R cells, consistent with its known functions.^[^ ^41^ ^]^

As GPx4 regulates lipid‐dependent anti‐oxidant responses, we next examined oxidized membrane lipid levels in OVCAR5 cells transfected with shRNA targeting GPx4 versus shCTRL. Staining with C11 BODIPY showed increased oxidized membrane lipid levels in GPx4 KD compared with control cells (Figure 5E), confirming its role in preventing ferroptosis. To determine whether intracellular cholesterol levels could rescue ferroptosis under conditions of GPx4 depletion, Pt‐R OVCAR5 cells under basal conditions (1% FBS) were provided with free cholesterol (50 µg mL^−1^, 48 h). Exogenous cholesterol reduced the increased oxidized lipids in GPx4 KD Pt‐R OVCAR5 compared with control cells (Figure 5F). Additionally, Pt‐R OC cells depleted of GPx4 were less viable under low serum conditions compared with control cells. Addition of exogenous cholesterol rescued GPx4 KD cells (Figure 5G), confirming the dependence of Pt‐R cells on cholesterol and GPx4. Likewise, exogenous cholesterol rescued viability in OVCAR5 Pt‐S (Figure 5H) and Pt‐R cells (Figure 5I) treated with the GPx4 inhibitor, RSL‐3, supporting that cholesterol uptake can buffer the toxic effects of increased intracellular ROS. In contrast, inhibition of cholesterol uptake through SR‐B1 blockade by using HDL NP induced an increase in ROS levels (Figure 5J).

To further support the connection between SR‐B1 and GPx4, we examined the effects of genetic KD of SR‐B1 on GPx4 expression level. SR‐B1 depletion by siRNA led to a decrease in GPx4 expression as determined by western blot (Figure 5K,L). Combined, these results support that GPx4‐mediated cellular redox homeostasis in OC cells is linked to SR‐B1 expression levels and cholesterol accumulation, providing a rationale for targeting cholesterol uptake in Pt‐R cancer cells to induce ferroptosis.

### 2.6 GPx4 Blockade Inhibits SREBF2 Mediated SR‐B1 Expression in OC Cells

**Figure 6:**
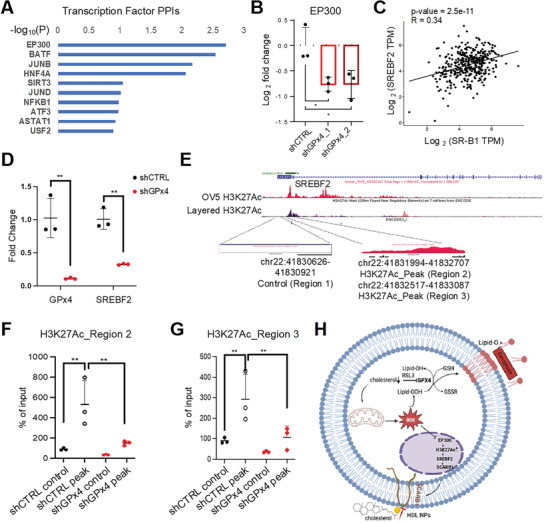
GPx4 knock down inhibits SR‐B1 expression through decreased H3K27Ac on SREBF2 promoter. A) Protein‐protein interactions (PPIs) analysis of top TFs binding targets in the downregulated DEGs of OVCAR5 Pt‐R cells transfected with shRNAs targeting GPx4 (shGPx4) versus shCTRL cells. Gene expression was measured by RNA‐seq (*n* = 3). B) Measurements by qRT‐PCR of EP300 mRNA in shGPx4 versus shCTRL OVCAR5 Pt‐R cells (mean ± SD, *n* = 3). Results include two shGPx4 sublines. C) A scatter plot shows the correlation between expression levels of *SCARB1* and *SREBF2* in HGSOC specimens from TCGA The Pearson's correlation coefficient (r) and *p*‐value are indicated (*n* = 370). D) GPx4 and SREBF2 mRNA expression in shGPx4 versus shCTRL OVCAR5 Pt‐R cells measured by qRT‐PCR (means ± SD, *n* = 3). E) UCSC Genome Brower on Human GRCh38/hg38 tracks of the H3K27Ac binding peak in the SREBF2 gene in OVCAR5 cells^[^ ^45^ ^]^ and other cell lines previously recorded in the ENCODE dataset (GM12878, and HEK293T).^[^ ^46^, ^47^ ^]^ The H3K27Ac binding motif indicated along with the position of primer sequences (Primer_2,3) used for q‐PCR are shown. Amplification of a sequence 1 kb downstream was used as a control (Primer_1). F,G) ChIP‐PCR shows H3K27Ac enrichment in the SREBF2 gene (primer_2 (F) and primer_3 (G)) in shCTRL versus shGPx4 OVCAR5 Pt‐S cells. Enrichment of H3K27Ac on a sequence 1 kb downstream was used as a control (Primer_1) (mean ± SD, *n* = 3). H) Model shows mechanism interconnecting cholesterol and anti‐redox pathway. For all panels, *p < 0.05; **p < 0.01; ***p < 0.001).

To further study the molecular mechanism by which increased GPx4 maintains high SR‐B1 expression and cholesterol uptake in OC cells, we examined GPx4 related transcriptomic signatures in Pt‐R OVCAR5 cells by comparing GPx4 KD versus control cells. Protein‐Protein Interaction (PPI) analysis identified top transcriptional regulators enriched among the downregulated DEGs in GPx4 KD OC cells. The histone acetyltransferase EP300 was the most significantly suppressed transcriptional regulator in GPx4 KD cells (**Figure** **6**A). We validated down‐regulation of EP300 expression in OVCAR5 Pt‐R cells in which GPx4 was KD through qRT‐PCR (Figure 6B).

Among the key TFs involved in regulating SR‐B1 expression and cholesterol metabolism are the sterol regulatory binding factors 1 and 2 (SREBFs, also referred to as SREBPs) encoded by the *SREBF 1* and *2* genes.^[^ ^42^, ^43^ ^]^ We found a strong correlation between the SR‐B1 and SREBF2 or 1 expression levels in HGSOC tumors profiled by TCGA (Figure 6C, *R* = 0.34, *p* = 2.5 e‐11; Figure S5B, Supporting Information, *R* = 0.26, *p* = 5e‐07). While the two related TFs have somewhat overlapping functions, SREBF2 was linked with regulation of key genes in cholesterol metabolism, while SREBF1 was shown to regulate genes in the fatty acid synthesis pathway^[^ ^44^ ^]^ and SREBF2 was found to be a more potent regulator of SR‐B1 transcription as compared with SREBF1.^[^ ^43^ ^]^ Therefore, in subsequent studies we focused on SREBF2. This TF was downregulated in OC cells in which GPx4 was KD (Figure 6D), suggesting a potential link between GPx4 and SREBF2.

To uncover the mechanism by which SREBF2 was downregulated in GPx4 KD cells, especially given the substantial inhibition of EP300 in these cells, we investigated potential epigenetic regulation of SREBF2 by EP300 in the GPx4 KD cells. Previous studies showed acetylation of H3K27 in the promoter region of the SREBF2 gene in OVCAR5 cells^[^ ^45^ ^]^ and other cell lines analyzed in the ENCODE dataset (GM12878 and HEK293T)^[^ ^46^, ^47^ ^]^ (Figure 6E and Figure S5C, Supporting Information), suggesting conserved regulation of the SREBF2 gene by H3K27Ac. Cut&Tag sequencing using an H3K27Ac antibody detected a reduction of H3K27Ac enrichment at the SREBF2 promoter in OC cells in which GPx4 was KD (hg38_dna range = chr22: 41 831600‐41833300, Figure 6E). ChIP‐qPCR using an antibody against H3K27Ac, with primers flanking the known H3K27Ac peak in the SREBF2 promoter (hg38_dna range = chr22:41831994‐41832707_region 2; chr22:41832517‐41833087_region 3; Figure 6E) confirmed H3K27ac enrichment in the SREBF2 promoter region compared with a control region (hg38_dna range: chr22:41830626‐41830921_region 1; Figure 6F,G), as well as reduction of H3K27ac enrichment in cells in which GPx4 was KD. In contrast, GPx4 KD had little impact on H3 enrichment on the promoter region of SREBF1 (Figure S5C, Supporting Information). Thus, H3K27Ac deposition enriched in the promoter region of SREBF2, was significantly inhibited in cells in which GPx4 was KD. These data support that OC cells in which GPx4 is KD, harbor increased ROS levels, decreased EP300 expression resulting in reduced histone acetylation on the SREBF2 promoter and suppressed SR‐B1 expression, as illustrated in Figure 6H.

## 3 Conclusion

The development of resistance to Pt‐based chemotherapy is challenging because of the lack of effective treatments for resistant cancers. The Pt‐R OC phenotype requires increased enzymatic detoxification of L‐ROS, which involves changes in fatty acid and cholesterol metabolism. Our data reveal that Pt‐R OC requires increased cholesterol uptake through SR‐B1 and increased activity of the glutathione (GSH)‐dependent GPx4 enzyme that reduces cell membrane L‐ROS. Knocking down GPx4 caused changes in SREBF2 activity, a key regulator of SR‐B1 expression. This regulatory axis was therapeutically targeted using a novel synthetic ligand of SR‐B1, HDL NP. We show that HDL NPs binding SR‐B1 in Pt‐R OC models inhibit cell cholesterol uptake and drastically reduce GPx4 expression, increase L‐ROS, and cause cell death through ferroptosis in vitro and in vivo. Therefore, targeting cell membrane anchored SR‐B1 and cholesterol uptake is a powerful way to disrupt redox pathways required for the survival of Pt‐R OC cells and tumors.

Biochemical and transcriptomic profiling reveal that Pt‐R OC cells harbor increased intracellular cholesterol and express higher levels of the transporter SR‐B1, while downregulating pathways related to de novo cholesterol biosynthesis. High expression of SR‐B1 in HGSOC was reported by others.^[^ ^14^, ^48^ ^]^ The data suggest increased reliance of Pt‐R cells upon crHDL binding SR‐B1 and support the concept that acquired changes in lipid metabolism are required cellular adaptations to reduce the toxicity induced by Pt and excess L‐ROS. This phenomenon is detectable in other cancers. Some lymphomas are auxotrophic for cholesterol because of hypermethylation and silencing or mutation of de novo cholesterol biosynthesis genes.^[^ ^40^ ^]^ Accumulation of cholesteryl esters has also been reported in PTEN‐null prostate cancer being associated with increased aggressiveness,^[^ ^13^ ^]^ and dependence on cholesterol uptake through SR‐B1 was described in clear cell renal cell carcinoma.^[^ ^35^ ^]^ The latter is notoriously resistant to chemotherapy and auxotrophic for cholesterol from exogenous sources. In fact, renal cell carcinoma demonstrates a “clear cell” pathophysiology due to reduced de novo cholesterol biosynthesis, uptake of HDL cholesterol through SR‐B1^[^ ^49^ ^]^ and cholesterol stored in lipid droplets.

It has been proposed that intracellular cholesterol and cholesterol‐pathway intermediates play critical roles in maintaining redox homeostasis and can protect cells from excess ROS,^[^ ^50^, ^51^ ^]^ but the mechanism is not clear. Cholesterol metabolism and accumulation of lipids have been shown to prevent cell death along with increasing the expression of GPx4, a potent antioxidant protein and inhibitor of ferroptosis.^[^ ^52^ ^]^ On the other hand, GPx4 inhibition was shown to induce cell death through a process dependent upon oxidized phospholipids (ferroptosis) which uniquely target treatment‐resistant cancer cells.^[^ ^8^, ^53^ ^]^ Examples of cholesterol uptake dependent cancers that are highly sensitive to targeted GPx4 inhibitors are clear cell renal carcinoma, lymphomas, and leukemias.^[^ ^35^ ^]^ Under certain circumstances, GPx4 can be inhibited by disruption of de novo cholesterol synthesis (e.g., using statins) resulting in increased ferroptosis. This is thought to occur because GPx4 contains selenocysteine residues which are activated during protein synthesis by isopentenyl‐5‐pyrophosphate, an intermediate of the mevalonate pathway of cholesterol synthesis.^[^ ^54^ ^]^

Here we show that targeting cholesterol uptake in Pt‐R OC models by using HDL NP decreased cancer cell viability and blocked tumor growth. A main mechanism of cytotoxicity in these OC models adapted to surviving under high ROS conditions was induction of ferroptosis. Flow cytometry experiments employing the membrane localizing C11‐BODIPY fluorophore reagent whose fluorescence is modified upon oxidation demonstrated that addition of HDL NPs results in a significant increase in cell membrane localized oxidation. Pt‐R OC cell death could be rescued by addition of the lipophilic cell membrane localizing antioxidant ferrostatin‐1 or the iron chelator deferoxamine which depletes iron rendering it unavailable as a co‐factor for lipid oxidation. Levels of GPx4 were significantly reduced in Pt‐R cells after treatment with HDL NP. Furthermore, by depleting GPx4, HDL NPs sensitized Pt‐R cells to carboplatin. These observations are consistent with prior results from our group testing HDL NP in lymphoma models.^[^ ^30^, ^39^, ^55^ ^]^

There is a strong relationship between enhanced uptake of cholesterol by chemotherapy resistant cancer cells and the requisite high expression of GPx4 to prevent lipid peroxidation and cell death by ferroptosis. Interestingly, prior data suggest that cholesterol modulation of cell membrane fluidity and lipid raft formation was important for modulating the diffusion of membrane localized substrates for oxidation and cell sensitivity to GPx4‐mediated ferroptosis.^[^ ^36^, ^56^ ^]^ These reports suggest the possibility that HDL NP induced ferroptosis is triggered by modulating membrane fluidity and lipid rafts to enhance cell membrane lipid peroxidation. Our past work supports that HDL NPs also enhance cholesterol efflux from the cell membrane after binding SR‐B1 and that this disrupts membrane lipid rafts.^[^ ^26^ ^]^ Thus, aside from restricting cholesterol uptake, HDL NPs could also reduce cell membrane cholesterol and modulate membrane fluidity and lipid rafts to facilitate ferroptosis. Here we show that replenishment of cholesterol stores in Pt‐R OC cells rescues ferroptosis induced by GPx4 knockdown or inhibition, consistent with this mechanism.

Accumulation of cholesterol in chemoresistant cancer cells, highly enriched in antioxidant molecules such as GPx4,^[^ ^8^ ^]^ remains unexplained. Here we show that GPx4 expression levels, which reduce intracellular ROS induced by exposure to chemotherapy, are directly correlated with cholesterol uptake and intracellular cholesterol stores. We propose epigenetic repression of SREBF2 as a potential mechanism by which GPx4 modulated redox balance in Pt‐R OC cells alters cholesterol uptake. SREBF2 is a transcription factor that directly regulates the expression of the HDL transporter SR‐B1 exerting more potent effects than the related factor SREBF1.^[^ ^43^ ^]^ Under high ROS levels caused by GPx4 knockdown, expression of the EP300 histone acetyltransferase is downregulated, leading to decreased histone acetylation at the SREBF2 promoter and reducing its expression levels. Regulation of EP300 by GPx4 and ROS levels is consistent with previous observations supporting that oxidative stress could alter epigenetic mechanisms regulating transcription.^[^ ^57^, ^58^, ^59^ ^]^ Our results establish a link between redox balance, histone acetylation, and cholesterol metabolism. We recognize that EP300 downregulation could have more global transcriptional effects.

Our data detail a pathway whereby Pt resistance is enabled by an increased cell membrane requirement for cholesterol through crHDL binding SR‐B1 and increased expression of the antioxidant enzyme, GPx4. A synthetic ligand of SR‐B1, HDL NP, that targets cell membrane cholesterol and the uptake of cholesteryl esters induces cell death by ferroptosis. Single‐agent efficacy is possible due to reduced cholesterol and cholesteryl ester delivery to the cell and reduced expression of GPx4. An epigenetic mechanism links the antioxidant protein, GPx4 to key regulators of cholesterol metabolism. We anticipate that the dual mechanism through which HDL NPs induce ferroptosis will be therapeutically relevant to other human cancers that are highly resistant to chemotherapy and radiation.

## 4 Experimental Section

### Human Specimens

Deidentified high grade serous ovarian tumors (HGSOC) were collected and processed fresh from consenting patients (Northwestern University IRB#: STU00202468). Human tumor tissues and OC xenografts were enzymatically disassociated into single cell suspensions and cultured as previously described (*n* = 6; see Supporting Information).^[^ ^8^, ^60^ ^]^

### Cell Culture

OVCAR5 cells were a generous gift from Dr. Marcus Peter, Northwestern University; OVCAR4 cells were from Dr. Mazhar Adli, Northwestern University; immortalized human fallopian tube epithelial cells (FT190) were from Dr. R. Drapkin of University of Pennsylvania;^[^ ^61^ ^]^ normal endometrial cells (NoEM)^[^ ^38^ ^]^ were from Dr. Serdar Bulun, Northwestern University, and IOSE cells were from Dr. N. Auersperg,^[^ ^62^ ^]^ University of British Columbia. Peo1 and Peo4 cells were purchased from Sigma Aldrich. Cells were maintained in a 37 °C incubator with 5% CO_2._ Low passage cells were used, and all cell lines were tested to be pathogen and Mycoplasma negative (Charles River Research Animal Diagnostic Services). Peo1 and Peo4 cells were cultured in RPMI‐1640 with L‐glutamine (Corning, Cat#10‐040‐CV) plus 10% FBS, 1% GlutaMAX (Gibco, Cat# 35050–061), 2 mM Sodium Pyruvate (Gibco, Cat# 11360‐070), and 1% penicillin‐streptomycin. OVCAR5 and OVCAR4 Pt‐S and Pt‐R cells were maintained in RPMI‐1640 with L‐glutamine (Corning, Cat# 10‐040‐CV) plus 10% FBS, 1% GlutaMAX, and 1% penicillin‐streptomycin. Primary patient tumor derived cells were maintained in DMEM with L‐glutamine, 4.5 g L^−1^ glucose, without sodium pyruvate (Corning, Cat#10‐017‐CV) plus 10% FBS, 1% GlutaMAX, and 1% penicillin‐streptomycin. Primary human fallopian tube epithelial cells (HFTEC) were purchased from Lifeline Cell Technology (Frederich, MD) and cultured in the complete ReproLife Reproductive Medium (Lifeline Cell Technology, Cat# LL‐0068). Pt‐R sublines of OVCAR4 and OVCAR5 OC cells were developed through repeated exposure to 3 or 4 equal or increasing doses of cisplatin or carboplatin for 24 h, as described previously (see Supporting Information).^[^ ^8^ ^]^

### Chemicals and Reagents

RSL3 was purchased from Fisher Scientific (Cat# 611 810). Atorvastatin was from Selleck Chem (Cat# S5715). Cisplatin (Cat# 1 134 357), carboplatin (Cat# C2538), and cholesterol (Cat# C3045) were from Sigma‐Aldrich.

### HDL NP Synthesis

HDL NPs were synthesized and quantified as described previously.^[^ ^27^, ^31^ ^]^ An aqueous solution of 5 nm diameter citrate stabilized gold nanoparticles (AuNP) (75 nm, Nanocomposix) was surface‐functionalized with purified human apolipoprotein A‐I (apoA‐I) (1.3 mg mL^−1^ MyBiosource, MBS135961, fivefold molar excess). The AuNP/ apoA‐I mixture was incubated on a flat bottom shaker at 60 rpm for 1 h at room temperature (RT). Next, 100% ethanol was added to AuNP/ apoA‐I mixture to bring the concentration of ethanol to 20%. Then two different phospholipids, 1,2‐dipalmitoyl‐*sn*‐glycero‐3‐phosphoethanolamine‐N‐[3‐(2‐pyridyldithio) propionate] (PDP PE) (Avanti Polar Lipids #870205P) and 1,2‐dipalmitoyl‐*sn*‐glycero‐3‐phosphocholine (DPPC) (Avanti Polar Lipids #850355P), both dissolved in ethanol (1 mM), were added to the solution at a final concentration that is 250‐fold molar excess to AuNP. PDP PE was added first in order to facilitate binding to the AuNP through the thiolate bond, and the DPPC solution was added second. These HDL NPs were incubated on a flat bottom shaker at 60 rpm overnight at RT followed by purification by tangential flow filtration (TFF: KrosFlo Research KRi2 TFF System, Repligen, model 900–1613). HDL NPs were stored at 4 °C until use. UV–vis spectroscopy (Agilent 9453) and Beer's Law were used to measure the concentration of HDL NPs where 5 nm AuNPs have a characteristic absorption at *λ*_max_ = 520 nm and extinction coefficient of 9.696 × 10^6^ m ^−1^ cm^−1^.

### Cell Viability Assay

Cell viability was evaluated using the Cell Counting Kit 8 assay (CCK8, Dojindo Molecular Technologies, Cat# CK04, Rockville, MD, USA) following the manufacturer's recommendations. Absorbances (450 nm) were measured with a microplate reader (BioTek ELX800, BioTeK, Winooski, VT). For experiments using HDL NPs, cell viability was measured with MTS assays (CellTiter, Promega, Madison, WI) as previously described.^[^ ^63^ ^]^ Cells were plated at 500 cells well^−1^ in 96‐well plates and treated with PBS or HDL NP for 72 or 96 h depending on cell line. For ferroptosis experiments, cells were additionally treated with a final concentration of 1 µm of ferrostatin‐1 or deferoxamine (DFO) (Sigma). For statin experiments, cells were treated with 2.5 or 5 µm atorvastatin (Cayman Chem). For synergy of carboplatin and HDL NPs, OVCAR5 Pt‐R cells were treated with 3.125, 6.25, 12.5, 25, 50, or 100 nm HDL NP and/or 3.125, 6.25, 12.5, 25, 50, or 100 µm carboplatin. IC_50_ values were calculated using GraphPad Prism and the combination index was calculated using the method of Chou‐Talalay.^[^ ^64^ ^]^

### Cisplatin Half Maximal Inhibitory Concentration (IC_50_)

Five thousand cells per well were seeded in 96‐well plates and treated with different concentrations of cisplatin: 0, 0.5, 1, 2.5, 5, 10, 25, 50, 75, 100, 250, and 500 µM for 24 h. Cell viability was measured at 72 h post‐cisplatin treatment by a CCK8 assay. IC_50_ values were calculated using GraphPad Prism software as described previously.^[^ ^8^ ^]^

### In Vivo Experiments

Animal studies were conducted according to protocol # IS00017063 approved by the Institutional Animal Care and Use Committee of Northwestern University. Two million Pt‐R OVCAR5 cells were injected intraperitoneally (i.p.) into female, 6–8 weeks old, nude mice (*Foxn1^nu^*, Envigo). 3 days after cell injection, mice were randomized and administered daily (Monday through Friday, 2 days off) i.p. treatments of 200 µL PBS (control) or HDL NP (1 µm) for 4 weeks. Mice were weighed biweekly and euthanized the day after the last treatment. Tumors were counted, measured and weighed. Tumors were enzymatically digested to obtain single cell suspension for lipid peroxidation analysis using C‐11 BODIPY staining. For the PDX model, fresh tumor tissue resected from subcutaneously engrafted ovarian PDX growing in NSG mice^[^ ^65^ ^]^ was sliced, minced, and quickly digested with 1.5 mg mL^−1^ collagenase IV (Millipore Sigma cat#C7657‐500MG), 50 µg mL^−1^ dispase (Thermo Fisher Scientific cat#17 105 041), 50 µg mL^−1^ liberase (Millipore Sigma cat#5 401 119 001), and 0.1 mg mL^−1^ DNase I (Thermo Fisher Scientific cat#EN0521) in Hanks Balanced Salt Solution (Thermo Fisher Scientific cat#88 284) in a shaker (Thermo Scientific MaxQ 8000 Incubated Stackable Shakers) at 250 rpm and 37 °C for 2 h. The digested tissue was passed through a 100 µm cell strainer (Corning Fisher Scientific cat#431 752) and the cell suspension was centrifuged, washed twice with Dulbecco's PBS and reconstituted for cell counting. The cell suspension was mixed with 20% matrigel. 2 × 10^6^ cells in 100 µL solution were injected i.p. using a 25 G needle. After 2 weeks, the 14 mice were randomly divided into two groups: control (PBS) and HDL NP (7 mice per group) and treated with either HDL NP (1 µm) or 1xPBS daily from Monday to Friday for 10 weeks. Body weights were monitored twice a week. All mice survived to the experimental end point (10 weeks). At necropsy, tumors were counted, measured, and weighed. All primary tumor tissue and organs were processed (fixed in 10% formalin, embedded in paraffin) and sectioned for histologic examination. Tumor histology was examined as previously described.^[^ ^66^ ^]^

### Large‐Area Hyperspectral SRS Imaging

Multiplex Stimulated Raman Scattering (SRS) was performed by a femtosecond laser with two synchronized outputs beams (Insight DeepSee, Spectra‐Physics, Santa Clara, CA, USA) and images was taken and analyzed using ImageJ as described previously (see Supporting Information).^[^ ^67^, ^68^ ^]^

### RNA Extraction and Quantitative RT‐PCR Analysis

Total RNA was isolated with Trizol (Invitrogen, Carlsbad, CA) following the manufacturer's instructions and quantitative RT‐PCR used the iTaq Universal SYBR Green Supermix (Bio‐Rad, Berkeley, California) and a 7900HT real‐time PCR instrument (Applied Biosystems, Foster City, CA) as previously described^11^ (see Supporting Information). Primer sequences (Integrated DNA Technologies, USA) are in Table S4, Supporting Information.

### ChIP‐PCR

ChIP was performed with anti‐H3 (Active Motif, Cat# 39 763), and anti‐H3K27Ac (Abcam, Cat# 4729) antibodies. Briefly, extracted chromatin was crosslinked with 1% paraformaldehyde and sonicated to an average size of ≈300–500 bp. 3 µgs of chromatin were incubated with 3 µg of either anti‐H3 or anti‐H3K27Ac overnight at 4 °C. The concentration of immunoprecipitated DNA was measured with a Qubit dsDNA HS Assay Kit (Thermo Fisher Scientific). Immunoprecipitated DNA was amplified by qPCR with gene‐specific primers using SYBR Green Master Mix (Bio‐Rad, iTaq Universal SYBR Green Supermix). A target sequence located 1 kb upstream from the binding site served as negative control. Normalization used input DNA. Primer sequences are listed in Table S5, Supporting Information.

### Western Blotting

Cells were lysed in radio immunoprecipitation assay (RIPA) buffer or mammalian protein extraction reagent (mPER, ThermoFisher, Waltham, MA). Protein concentrations were quantified with the Bradford assay (Biorad Protein Assay Reagent, BioRad, Berkeley, CA) or BCA assay (Pierce BCA Protein Assay Kit, ThermoFisher, Waltham, MA). Proteins (10–20 µg) were resolved by SDS‐PAGE, and electroblotted onto PVDF membranes, as described previously.^[^ ^8^ ^]^ The antibodies against GPx4 (rabbit monoclonal, Cat# ab125066, used at 1:1000), and scavenging receptor SR‐B1 (rabbit monoclonal, Cat# 52 629, used at 1:1000) were purchased from Abcam (Cambridge, MA). HMGCS1 (rabbit monoclonal, Cat# 36 877, 1:1000), SQLE (rabbit monoclonal, Cat# 40 659, 1:1000), and β‐actin (rabbit monoclonal, Cat# 4970) were from Cell Signaling Technology (Danvers, MA). Mouse monoclonal GAPDH antibody was from Meridian Life Science (Memphis, Tennessee, Cat# H86504M, used at 1:10000). Mouse monoclonal alpha tubulin (Rosemont, IL, Cat# 66031‐1, used at 1:100000) was from Proteintech. HRP‐conjugated donkey‐anti‐rabbit polyclonal antibody (Cat#NA9340, used at 1:2000) was purchased from GE Healthcare (Pittsburgh, PA), HRP‐conjugated goat‐anti‐mouse antibody (Cat#haf007, used at 1:2000) was from R&D System (Minneapolis, MN), and Horseradish Peroxidase (HRP)‐conjugated goat‐anti‐rabbit (Cat#1 706 515, used at 1:2000) was from BioRad (Berkely, CA). After blocking, membranes were incubated with primary antibody at 4 °C overnight and with HRP‐conjugated secondary antibody for 1 h at room temperature. Signal was generated using SuperSignal West Pico PLUS Chemiluminescent Substrate (Thermo Scientific, Cat# 34 577), SuperSignal West Femto Maximum Sensitivity Substrate (Thermo Scientific, Cat# 34 095) enhanced chemiluminescent HRP system, or Clarity Western ECL Substrate (BioRad, Berkely, CA). Images were captured by a luminescent image analyzer with a CCD camera (LAS 3000, Fuji Film) or on an Azure300 system (Azure Biosystems, Dublin, CA).

### Cell Transduction And Transfection

OC cells were transduced with lentiviral particles containing shRNA in the presence of polybrene (8 µg mL^−1^) for 48 h. Lentiviral transduction particles containing three shRNAs targeting GPx4 were used (shGPx4‐1, Cat#TRCN0000046249; shGPx4‐2, Cat#TRCN0000046251, and shGPx4‐3, Cat#00 00046252). Cells transduced with scrambled shRNA (Mission Lentiviral Transduction Particles, Sigma‐Aldrich, St Louis, MO, USA) served as controls. Transduced cells were selected with puromycin (2 µg mL^−1^). For transient siRNA transfection, cells were transfected with 25 nm siRNAs against SR‐B1 (Cat# AM16708, Invitrogen) or control siRNAs (Cat# AM4611, Invitrogen) using RNAiMax (Thermo Fisher). Cells mock transfected with lipofectamine alone were used as a control. Cells were evaluated 72 h after transfection.

### Intracellular Reactive Oxygen Species

Mean ROS levels were measured by using the DCFDA/H2DCFDA‐Cellular ROS Detection Assay Kit (Abcam, Cambridge, MA, USA). For RSL3 treatment, 25, 000 cells per well were seeded in a dark, clear bottom 96‐well microplate, were treated with RSL3 or control and ROS levels were measured by fluoro‐spectrophotometer at Ex/Em wavelengths of 480 and 535 nm, respectively. Cells transduced with shRNAs (shCTRL or shGPx4) were cultured in full serum (10% FBS) for 24 h. A million cells were treated with 20 µm DCFDA for 30 min at 37 °C prior to FACS analysis.

### BODIPY Staining for Lipid Peroxidation

Intracellular lipid peroxidation was determined with BODIPY 581/591 C11 (Thermo Fisher Scientific, Cat# D3861), a lipid peroxidation sensor as previously described^[^ ^11^ ^]^ (see Supporting Information). Data were analyzed using FlowJo software.

### Intracellular Cholesterol Quantification Assay

Total intracellular cholesterol, including free cholesterol and cholesteryl ester, was measured using an Amplex red cholesterol assay kit (Cat# A12216, Thermal Fisher Science, USA), as described previously.^[^ ^37^ ^]^ Briefly, cells cultured in full serum conditions were detached using trypsin, counted, and lysed in RIPA buffer or directly in 1X Reaction Buffer. For quantification, 50 µL of standards or samples and 50 µL of the Amplex Red reagent/HRP/cholesterol oxidase/ cholesterol esterase working solution (300 µm Amplex Red reagent containing 2 U mL^−1^ HRP, 2 U mL^−1^ cholesterol oxidase, and 0.2 U mL^−1^ cholesterol esterase) were added to a 96‐well black plate and incubated for 30 min at 37 °C in the dark. Fluorescence intensities were measured in a microplate reader using Ex/Em of 560/590 nm. Cholesterol content was normalized to cell number (per 10^5^ cells mL^−1^).

### Intracellular Lipid Quantification

Intracellular lipids were quantified using Nile Red reagent (Sigma Aldrich). Cells were plated at 150, 000 cells per well in 6‐well plates. The following day, cells were incubated with Nile Red (300 nm) for 15 min at 37 °C, 5% CO_2_, washed twice with 1X PBS, and resuspended in 1X PBS Fluorescence was quantified using a BD LSR II Fortessa flow cytometer and data were analyzed using FlowJo software.

### Triglyceride Assay

Cellular triglyceride levels were detected with the Triglyceride‐Glo Assay (Promega, Madison, WI) according to the manufacturer's protocol. Cells were plated at 20, 000 cells well^−1^ in 96‐well plates and allowed to attach to the plate overnight. Cells were washed twice with PBS, 50 µL lysis buffer with lipase was added to each well, and the plate was incubated at 37 °C for 30 min. 45 µL of sample, lysis buffer, or a 40 µm standard were added to a white 96‐well plate. 45 µL of detection reagent was added to each well and incubated at room temperature for 1 h. Luminescence was read on a Synergy 2 plate reader (BioTek).

### Cholesterol Uptake

Cells were plated at 100, 000 cell per well in 6‐well plates. The following day, media was changed to 1% FBS containing media and cells were incubated for 24 h. TopFlour (TF) cholesterol (1 µm, Avanti Polar Lipids) with or without HDL NPs (10, 20 or 40 nm) were added to cells and incubated for 4 h at 37 °C, 5% CO_2_. Cells were then washed twice with 1X PBS, resuspended in 1X PBS, and TF cholesterol fluorescence was quantified using a BD LSR II Fortessa flow cytometer in the FITC channel. Data were analyzed using FlowJo software.

### RNA Sequencing

Total RNA was extracted with TRI Reagent (Sigma, Cat# T9424), processed as previously described and sequenced on an Illumina NextSeq500 system with single‐end, 75‐bp read length settings (See Supporting Information). Data are deposited in the NCBI GEO database (GSE234404).

### Data Analysis

Normalized read counts from RNA sequencing of Pt‐S and Pt‐R OVCAR5 cells (GSE 148 003)^[^ ^8^ ^]^ were used for GSEA^[^ ^69^ ^]^ by using Hallmark and C2 curated gene sets. Heatmap for Hallmark_Cholesterol_Homeostasis gene set was generated by using the heatmap R package. BioJupies https://www.cell.com/cell‐systems/fulltext/S2405‐4712(18)30432‐0 was used to analyze pathways enriched among differentially expressed genes (DEGs) in cells depleted of GPx4 versus control cells. Transcription factor PPI pathway enrichment used Enrichr https://maayanlab.cloud/Enrichr/. RNA sequencing data obtained from HGSOC tumors were downloaded from The Cancer Genome Atlas (TCGA‐OV). Linear correlation analysis was performed using *n* = 370 samples to test the relationship between genes of interest. Pearson's correlation coefficient was calculated based on normalized counts. For Kaplan‐Meier survival analysis, an online tool was used (https://kmplot.com/analysis/) and a database including gene expression data and overall survival information of patients with HGSOC.^[^ ^70^ ^]^ A total of 523 samples from GEO were analyzed, including GSE14764, GSE15622, GSE18520, GSE19829, GSE23554, GSE26193, GSE26712, GSE27651, GSE30161, GSE3149, GSE51373, GES63885, GSE65986, GSE9891 and TCGA. The log rank test determined the statistical significance of survival differences between high versus low SCARB1 groups.

### Statistical Analysis

Data were analyzed by Student's *t‐*test, one‐way ANOVAs with Tukey's multiple comparisons test, or two‐way ANOVAs with Sidak's multiple comparisons test. *p*‐values from ANOVAs are multiplicity adjusted *p*‐values. All experiments were done in at least biological triplicates. All statistical analyses were done using GraphPad Prism software. For all experiments, *p* values less than 0.05 were considered significant. Outliers were selected by using Outlier Calculator (GraphPad) and a significance level of 0.05.

## Conflict of Interest

CST and AEC have a relationship with Zylem Biosciences, Inc., which is a start‐up biotechnology company with license to the HDL NP technology from Northwestern University.

## Supporting information

Supporting Information

## Data Availability

The data that support the findings of this study are available in the supplementary material of this article.
