# Supplementary material for: Nanoparticle Targeting in Chemo‐Resistant Ovarian Cancer Reveals Dual Axis of Therapeutic Vulnerability Involving Cholesterol Uptake and Cell Redox Balance
Source: Adv Sci (Weinh). 2024 Jan 23;11(13):2305212. doi: 10.1002/advs.202305212 (PMC10987123; doi:10.1002/advs.202305212)
Supplement: Supplementary file 1 — Supporting Information [file ADVS-11-2305212-s001.pdf]

ADVANCED SCIENCE
Open Access

# Supporting Information

for *Adv. Sci.*, DOI 10.1002/advs.202305212

Nanoparticle Targeting in Chemo-Resistant Ovarian Cancer Reveals Dual Axis of Therapeutic Vulnerability Involving Cholesterol Uptake and Cell Redox Balance

*Yinu Wang, Andrea E. Calvert, Horacio Cardenas, Jonathon S. Rink, Dominik Nahotko, Wenan Qiang, C. Estelle Ndukwe, Fukai Chen, Russell Keathley, Yaqi Zhang, Ji-Xin Cheng, C. Shad Thaxton* and Daniela Matei**

Supporting Information

**Nanoparticle Targeting in Chemo-Resistant Ovarian Cancer Reveals Dual Axis of Therapeutic Vulnerability Involving Cholesterol Uptake and Cell Redox Balance**

*Yinu Wang, Andrea E. Calvert, Horacio Cardenas, Jonathon S. Rink, Dominik Nahotko, Wenan Qiang, C. Estelle Ndukwe, Fukai Chen, Russell Keathley, Yaqi Zhang, Ji-Xin Cheng, C. Shad Thaxton*, Daniela Matei**

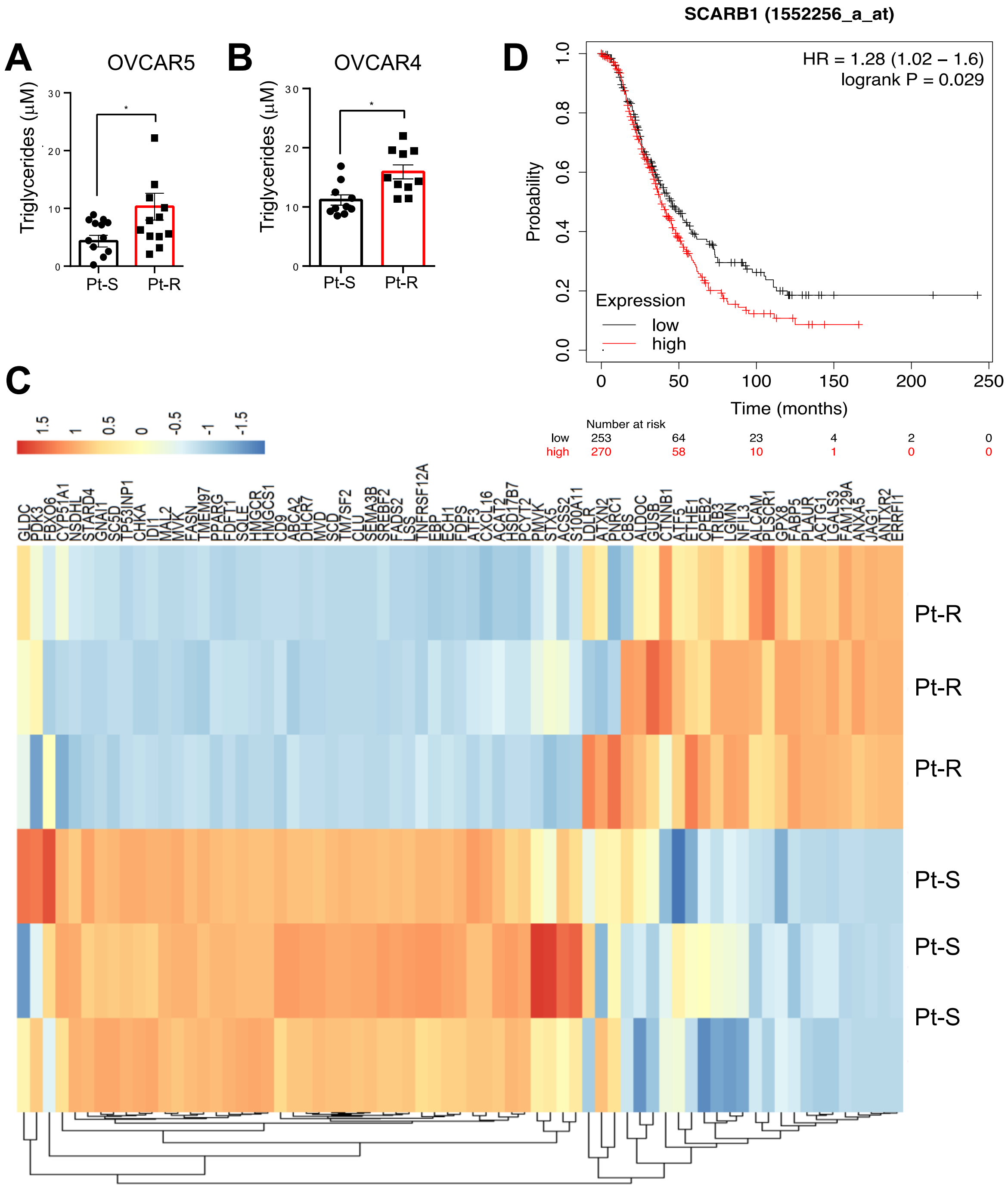


**Figure S1. (A, B)** Average triglyceride content in Pt-S and Pt-R OVCAR5 (**A**) and OVCAR4 **(B)** cells as measured by the Triglyceride-Glo™ Assay (n ≥ **10,** *p<0.05, **p<0.01). **(C)** Hierarchical clustering heatmap for differentially expressed (FDR<0.05) GSEA Hallmarks Cholesterol Homeostasis genes in OVCAR5 Pt-R vs. Pt-S OC cells. Gene expression was measured by RNA- seq (n=3 replicates/group). **(D)** Kaplan-Meier plot shows overall survival of HGSOC patients with high (n = 270) and low (n = 253) SCARB1 mRNA expression levels obtained from the TCGA and several DEO databases as described in Materials and Methods (n = 523), Survival curves were ploted by using KM Plotter.

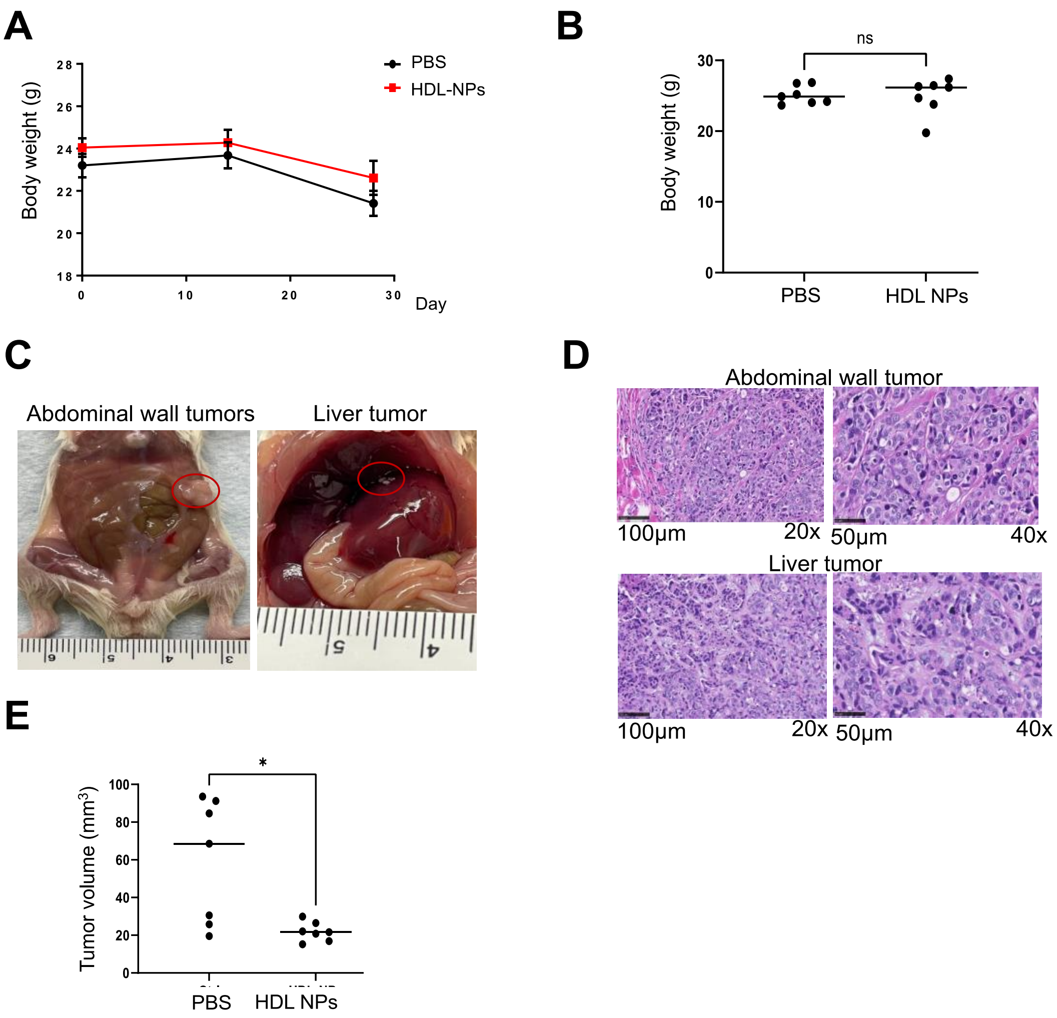


**Figure S2. (A, B)** Body weights of mice injected intraperitoneally (i.p.) with OVCAR5 Pt-R OC cells **(A,** n=17/group**)**, or OC Pt-R PDX cells **(B,** n=7/group**)** and treated with PBS or HDL NPs. **(C)** Representative images of i.p. OC Pt-R PDX tumors treated with PBS **(D)** H&E staining of i.p. OC Pt-R PDX tumors collected from the abdominal wall and liver. **(E)** Volumes of i.p. OC PDX tumors obtained from mice treated with PBS or HDL-NPs (5 days/week, for 10 weeks) (n=7 mice per group). Values are means ± SD, *p<0.05.

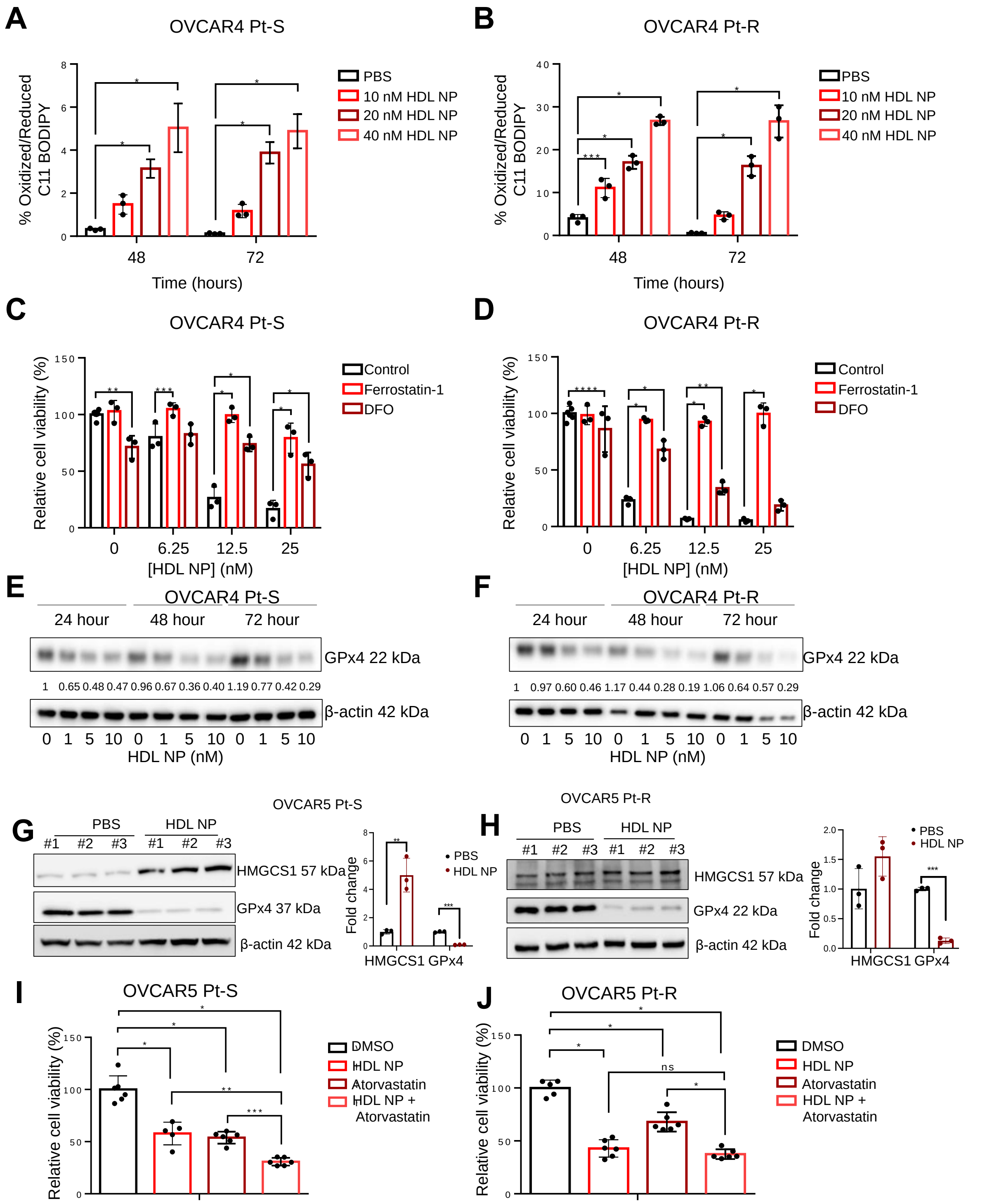


**Figure S3. (A, B)** OVCAR4 Pt-S **(A)** and Pt-R **(B)** cells were treated with HDL NPs for 48 or 72 hrs and percentage of oxidized/reduced lipids analyzed by C11 BODIPY staining (mean ± SD, n = 3). **(C, D)** OVCAR4 Pt-S **(C)** and Pt-R **(D)** cells were treated with HDL NP with or without ferrostatin-1 or DFO and cell viability assessed by MTS assay as a percentage of control (mean ± SD, n = 3). **(E, F)** Western blot analysis of GPx4 and β-actin (loading control) protein levels in OVCAR4 Pt-S **(E)** and Pt-R **(F)** cells treated with HDL NP for 24, 48, or 72h. Band intensities were quantified by densitometric analysis, results are shown below each blot. **(G, H)** **(Left)** Western blot analysis of HMGCS1 protein expression in OVCAR5 Pt-S **(G)** and Pt-R cells **(H)** treated with PBS and HDL NP (40 nM, 48 hours, n=3 replicates). (Right) Quantification and statistics of band intensities are shown in the bar graph. **(I, J)** Cell viability

assay of OVCAR5 Pt-S **(I)**, and Pt-R **(J)** OC cells treated with PBS, HDL NPs (12.5 nM), atorvastatin (2.5 or 5 μM), or HDL NP + atorvastatin, n=5 replicates). For all panels, *p<0.0001, **p<0.001, ***p<0.01, ****p<0.05.

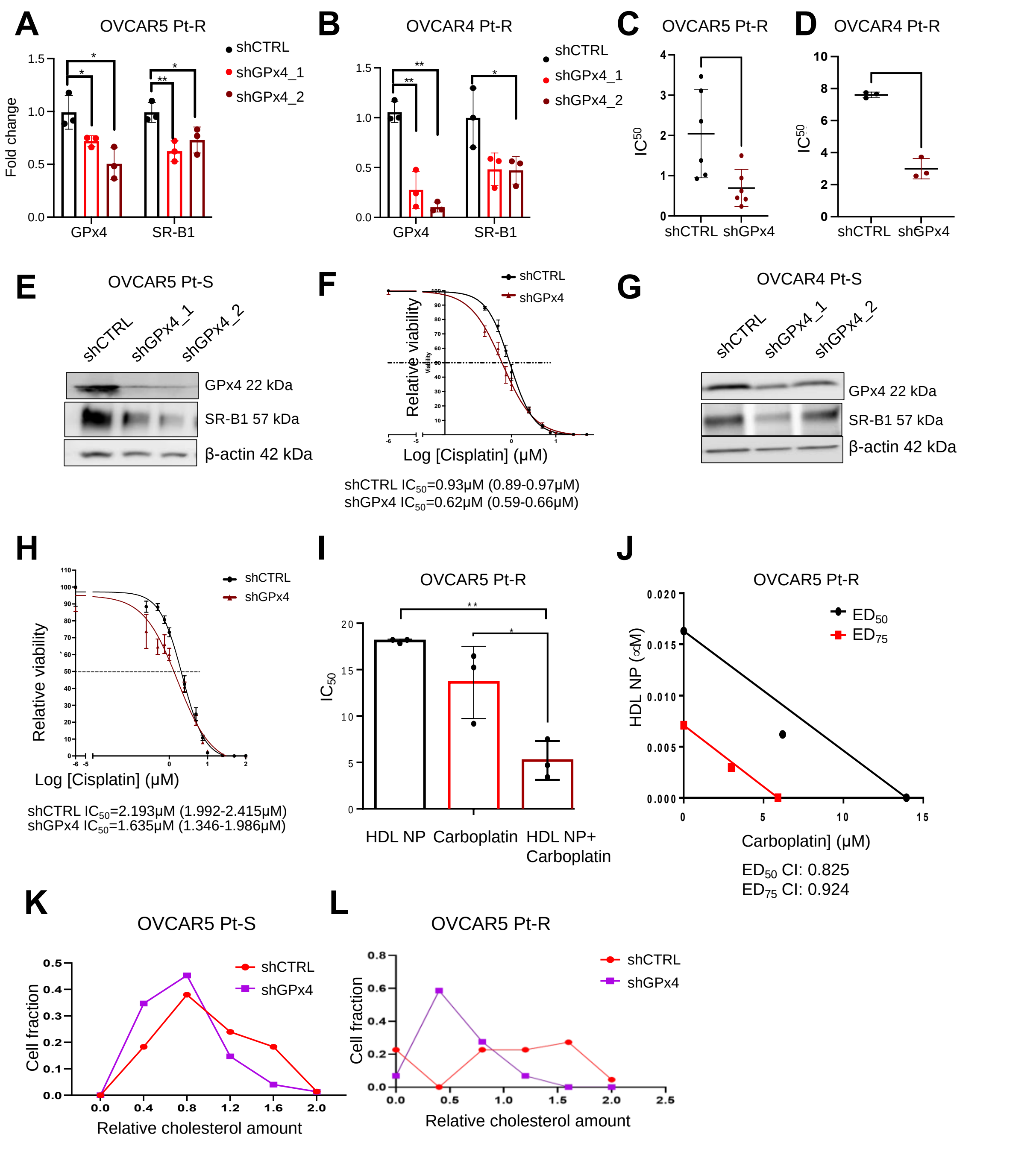


**Figure S4. (A, B)** Quantification by densitometric analysis of western blot bands of GPx4, SR-B1, and β-actin (loading control) in OVCAR5 Pt-R and OVCAR4 Pt-R cells shown in Figure 4 A-B. (**C**) Mean ± SD of half maximal inhibitory concentration ($IC_{50}$) of cisplatin effects on viability of OVCAR5 Pt-R cells transduced with shCTRL or shGPx4 (n=6 replicates). (**D**) $IC_{50}$ (mean ± SD) of cisplatin effects on viability of OVCAR4 Pt-R cells transduced with shctrl or shGPx4 (n=3 replicates). **(E, G)** SR-B1, GPx4 and GAPDH protein levels in shGPx4 and

shCTRL OVCAR5 **(E)** and OVCAR4 **(G)** Pt-S OC cells (n=3) **(F, H)** Cell viability assay measures survival of OVCAR5 **(F)**, OVCAR4 **(H)** Pt-S OC cells transfected with shGPx4 or shCTRL (mean ± SD, n = 3-4) after treatment with cisplatin (various concentrations). $IC_{50}$ are shown below. (**I, J**) $IC_{50}$ (mean ± SD) of HDL NP (3.125 - 100 nM), carboplatin (3.125 - 100 µM), or HDL NP (3.125 - 100 nM) + carboplatin (3.125 - 100 µM) effects on cell viability after treatment for 72 hrs (n = 3 replicates) in OVCAR5 Pt-R cells. **(J)** The combination indices (CI) of HDL NP + carboplatin effects on cell viability were calculated at $ED_{50}$ and $ED_{75}$ using Compusyn software. **(K,L)** Quantification of the SRS cholesterol channel signal intensity from OVCAR5 Pt-S and Pt-R OC cells at cell fraction level.

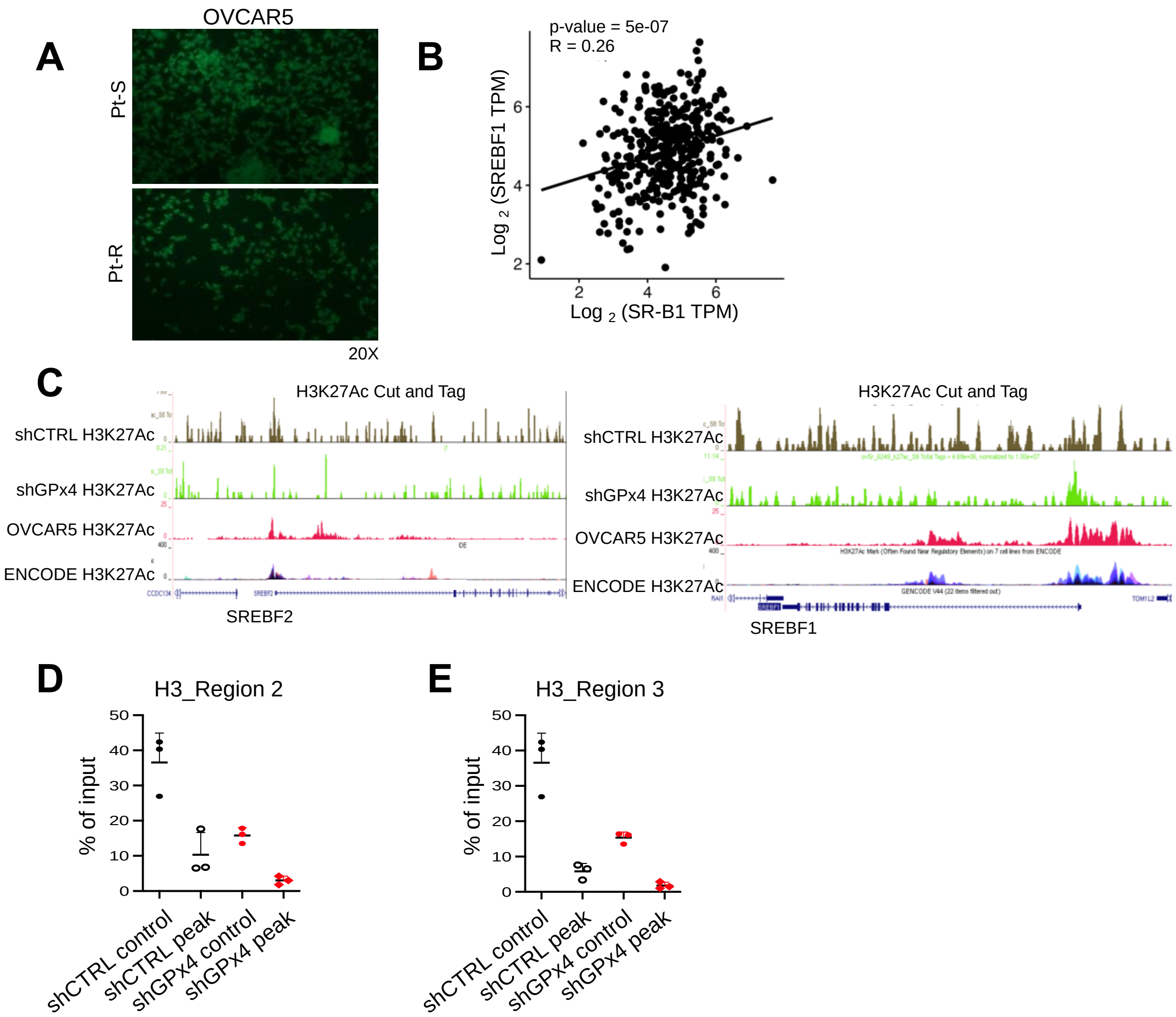


**Figure S5.** (**A**) Representative images of intracellular ROS in OVCAR5 Pt-S and Pt-R OC cells. **(B)** A scatter plot shows the correlation between expression levels of *SCARB1* and *SREBF1* in HGSOC specimens from TCGA The Pearson's correlation coefficient (r) and p-value are indicated (n = 370). **(C)** Gene enrichment peaks were measured by Cut&Tag (n=1 replicate per condition). The bottom two tracks are H3K27Ac binding peaks for SREBF2 (left), and SREBF1 (right) in OVCAR5 cells [45] and other cell lines previously recorded in the ENCODE dataset (GM12878, and HEK293T) [46, 47]. (**D**, **E**) ChIP-qPCR shows binding of H3 to the SREBF2 gene (primer_2 (**D**) and primer_3 (**E**)) in OVCAR5 Pt-S transfected with shCTRL vs. shGPx4 vectors. Enrichment of H3K27Ac on a sequence 1 kb downstream was used as a control (Primer_1) (mean ± SD, n = 3). For all panels, * p<0.05; **p<0.01; ***p<0.001.

**Table S1.** P and q values for the three selected significantly enriched gene sets

| Gene sets | Normalized Enrichment Score (NES) | p value | q value |
|---|---|---|---|
| Reactome_cholesterol_biosynthesis | -2.034 | 0.0 | 3.45E-4 |
| Hallmark_cholesterol_homeostasis | -1.700 | 0.0013 | 0.052 |
| Reactome_regulation_of_cholesterol_biosynthesis_by_SREBP_SREBF | -1.915 | 0.0013 | 0.0031 |

**Table S2.** List of transcripts that drive the three selected significantly enriched genesets

| Genesets | Transcripts |
|---|---|
| Reactome_cholesterol_biosynthesis | CYP51A1, GGPS1, NSDHL, MVD, PLPP6, ACAT2, LBR, HSD17B7, FDPS, FDFT1, LSS, SQLE, MVK, IDI1, HMGCR, SC5D, DHCR7, DHCR24, MSMO1, HMGCS1, TM7SF2 |
| Hallmark_cholesterol_homeostasis | CYP51A1, NSDHL, TNFRSF12A, TMEM97, ABCA2, MVD, CXCL16, ECH1, ACAT2, SREBF2, HSD17B7, FDPS, CHKA, FASN, STARD4, PPARG, FDFT1, LSS, FADS2, SQLE, MVK, ATF3, IDI1, HMGCR, SC5D, GNAI1, TP53INP1, DHCR7, HMGCS1, SCD, CLU, TM7SF2, SEMA3B, MAL2 |
| Reactome_regulation_of_cholesterol_biosynthesis_by_SREBP_SREBF | CYP51A1, GGPS1, TBL1X, MVD, SREBF2, CREBBP, FDPS, ACACA, SREBF1, FASN, FDFT1, ELOVL6, SMARCD3, LSS, SQLE, MVK, IDI1, HMGCR, SC5D, DHCR7, ACACB, HMGCS1, INSIG1, SCD, TM7SF2 |

**Table S3.** Patients' Characteristics.

| | Tumor Type | Treatment History | Sites |
|---|---|---|---|
| Pt #1_Pt83 | high grade serous carcinoma (stage IIIC) | chemotherapy-naïve | ovary |
| Pt #2_Pt96 | high grade serous carcinoma (stage IIIC) | total of 6 cycles of neoadjuvant chemotherapy with carboplatin/taxol/bevacizumab from 4/2021 to 7/2021 | uterus tumor; omentum tumor |
| Pt #3_Pt_94 | high grade serous carcinoma (stage IIIB) | chemotherapy-naïve | ovary |
| Pt #4_Pt99 | high grade adenocarcinoma (stage IIB, FIGUREO grade 2) | chemotherapy-naïve (BUT had chemothearpy to treat breast cancer in 2017) | ovary |
| Pt #5_Pt120 | high grade serous carcinoma (stage IIIC) | 4 cycles of carboplatin/taxol from 9/30/2021 | ovary |
| Pt #6_Pt114 | clear cell adenocarcinoma (stage IIIC) | carboplatin/taxol/bevacizumab Day 1, Cycle 1 from 12/16/2021 | omentum |

**Table S4.** Primer sequences for selected genes in qRT-PCR analysis.

| Gene Name | Primer Sequence | |
|---|---|---|
| | Forward (5' to 3') | Reverse (5' to 3') |
| 18S | CGT CTG CCC TAT CAACTTTC | GATGTGGTAGCC GTTTCTC |
| Ep300 | GCAGTGTGCCAAACCAGATG | GGGTTTGCCGGGGTACAATA |
| GPX4 | TCAGCAAGATCTGCGTGAAC | GGGGCAGGTCCTTCTCTATC |
| SREBF2 | TGAGCCAGGAAGCCCTCTAT | CAGAAGAATCCGTGAGCGGT |

**Table S5. Primer sequences for H3K27Ac ChIP-qPCR.**

| Gene Name | Primer Sequence | |
|---|---|---|
| | Forward (5' to 3') | Reverse (5' to 3') |
| SREBF2_Ctrl_Region 1_Primer_1 | CCAAATCTCACTACTCCTGTAAC | AGGAGACTGCGTCTCAAA |
| SREBF2_Ctrl Region 1_Primer_2 | CAATGTTGTCTTGCTTTCCC | TTGGCATTTCAGTATTGCTT |
| SREBF2_Peak_ Region 2_Primer_2 | TGAGGACCACTGGAGTAAG | TCCCAGCCTCCTTACAAA |
| SREBF2_Peak_ Region 2_Primer_2 | GGTATTCCATCGTGTGGATG | CAGCTGGTCGTGTTGTAAA |
| SREBF2_Peak_ Region 3_Primer_3 | GCCTGTTAACCCTTCACTC | CGTCCGGTCATCATCTTAAC |
| SREBF2_Peak_ Region 3_Primer_3 | TGCAAAGCAGAAGACGTAAA | TCCCAGCCTCCTTACAAA |

*Supplemental Materials and Methods:*

*Human specimens:* Tumors were minced into small pieces and digested with collagenase I (300 IU/ml, Sigma-Aldrich, Cat#C7657) and hyaluronidase (300 IU/ml, Sigma-Aldrich, Cat# H3506)) at 37 °C for 3 hours. Tumor suspensions were passed several times through a 16-18G needle, washed with PBS, and treated with red blood cell lysis buffer (BioLegend, Cat#420301) and DNase I (Qiagen, Cat# 79254). Lastly, cell suspensions were filtered through a 40 μm strainer (Fisher Scientific, Cat#NC0147038) to yield single- cell suspension, as previously described.[8, 60]

*Pt-R sublines of OVCAR4 and OVCAR5 OC cells* were developed through repeated exposure to 3 or 4 equal or increasing doses of cisplatin or carboplatin for 24 hours. Surviving cells were allowed to recover for 3 to 4 weeks before receiving the next treatment. Repeated exposure of OC cells to platinum induced a stable phenotype, with a least 2-fold increase in cisplatin or carboplatin $IC_{50}$, as described previously.[8]

*Large-area hyperspectal SRS imaging*: Multiplex Stimulated Raman Scattering (SRS) was performed by a femtosecond laser with two synchronized outputs beams (Insight DeepSee, Spectra-Physics, Santa Clara, CA, USA). The laser was operated at 80 HMz. One of the synchronized output beams, Pump beam, was fixed at 798 nm with the other beam, Stokes beam, being at 1040 nm to cover the C-H vibration region. An acousto-optic modulator (AOM, 1205-C, Isomet) was used to modulate Stokes beam at 2.3MHz. The SRS spectrum was recorded by controlling the temporal delay of two chirped femtosecond pulse. Six 12.7 cm long SF57 glass rods were applied to chirp both beams before the beams were sent to the laser-scanning microscope. A 60x water immersion objective lens (NA = 1.2, UPlanApo/IR, Olympus) was applied to focus the light on the sample with signal collection by applying an oil condenser (NA = 1.4, U-AAC, Olympus).[9, 67] To achieve hyperspectral SRS imaging, a stack of 100 images at various pump-Stokes temporal delay was recorded. To achieve large-area mapping, a motorized scanning stage (PH117, Prior Scientific) was applied.[68] The power of pump beam and Stokes beam were set to 30 mW and 200 mW, respectively. Raman shift calibration was completed by recording Raman spectrum of DMSO. Images were analyzed by ImageJ and least absolute shrinkage and selection operator (LASSO) was applied to separate different chemical maps according to their Raman spectrum. The reference of needed chemical maps were obtained by recording Raman spectrum of respective standard chemicals.[67]

*RNA sequencing (RNA-seq):* Total RNA was extracted with TRI Reagent (Sigma, Cat# T9424) and contaminating DNA was removed by using the RNeasy MinElute Cleanup Kit (QIAGEN) with RNase-Free DNase Set (QIAGEN). mRNA was isolated from 1 µg of total RNA with a NEBNext Poly(A) mRNA Magnetic Isolation Module. RNA sequencing libraries were prepared by using the NEBNext Ultra II RNA library prep kit and protocol from Illumina (New England Biolabs Inc., Ipswich, MA). After checking library quality with a BioAnalyzer (Agilent Technologies), libraries were sequenced on an Illumina NextSeq500 system with single-end, 75-bp read length settings. For quality control, raw fastq files were pre-processed using TrimGalore (0.4.4) and cutadapt (1.14) with single-end trimming mode, Phred score cutoff of 20 and minimum sequence length cutoff of 20 bp.[71] Quality was verified with the FastQC tool and raw sequencing reads were aligned to the human genome build hg38 using STAR v.2.5.2 (https://github.com/alexdobin/STAR). Mapped reads were converted to raw counts with HTSeq (https://htseq.readthedocs.io/en/master/), normalized to library size, and analyzed for DEGs by edgeR (Bioconductor). The $\log_2$(fold-change) and *P*- value of total normalized counts and DEG counts were then calculated. Data are deposited in the NCBI GEO database (GSE234404).

*RNA extraction and quantitative RT-PCR analysis:* Total RNA was isolated with Trizol (Invitrogen, Carlsbad, CA) following the manufacturer's instructions. RNA quantity and purity (260/280 absorbance ratio) were measured by using a NanoDrop spectrophotometer (Thermo Scientific). For *mRNA* expression studies, 0.2 to 1 µg of total RNA was reverse-transcribed into cDNA with an iScript cDNA synthesis kit (Bio-Rad, Berkeley, California). Quantitative RT-PCR used the iTaq Universal SYBR Green Supermix (Bio-Rad, Berkeley, California) and a 7900HT real-time PCR instrument (Applied Biosystems, Foster City, CA). The RT-PCR reaction used the following parameters: 94 °C for 10 min, 40 cycles of amplification at 94 °C for 15 s and 60 °C for 1 min, and an extension step of 7 min at 72 °C. Data were normalized using 18S gene as a control. Relative expression of target genes was calculated using the $2^{-\Delta(\Delta C_T)}$ method where $\Delta C_T = C_{T,\ target} - C_{T,\ 18S}$ and $\Delta(\Delta C_T) = \Delta C_{T,\ stimulated} - \Delta C_{T,\ control}$. Primer sequences (Integrated DNA Technologies, USA) are in **Table S4**.

*CUT&TAG:* CUT&Tag sequencing was performed with CUT&Tag-IT Assay Kit (Active motif, 53160) by following the manufacturer's protocol. Briefly, $10^5$ OVCAR5 Pt-R shCTRL and shGPx4 cells were collected and incubated with Concanavalin A beads for binding. Cells were then incubated with primary antibody targeting H3K27Ac (Cat#39034) overnight at 4 °C.

Samples were then incubated with secondary antibody, followed by CUT&Tag-IT Assembled pA-Tn5 Transposomes. After tagmentation at 37 °C for 1 hour, DNA was purified and cleaned up by using SPRI beads. DNA was then amplified with a combination of i7 and i5 Indexed primers for library preparation. Libraries were sequenced on Illumina HiSeq 4000 sequencer. Raw sequencing data were converted into fastq files which were checked by FastQC. Raw pair-end 50bp sequencing reads were mapped to human genome build hg38 using BoPt-Sie2 (v.2.2.6) with standard settings. Peaks were called using MACS2 (2.1.0) callpeak with cut-off FDR (q-value) at 0.05. Visualization was performed by IGV Genome Browser.

*BODIPY staining for lipid peroxidation:* Intracellular lipid peroxidation was determined with BODIPY 581/591 C11 (Thermo Fisher Scientific, Cat# D3861), a lipid peroxidation sensor. In brief, shCTRL or shGPx4 OVCAR5 cells were incubated with BODIPY 581/591 C11 (5 μM) at 37°C for 1 hr, washed with PBS, and fixed with 4% PFA on ice for 30 mins. OVCAR4 or OVCAR5 cells treated with HDL NPs (1, 5, 10, 20, or 40 nM) for 24, 48, or 72 hrs were incubated with BODIPY 581/591 C11 (2.5 μM) at 37°C for 1 hr and washed with PBS. In the cholesterol rescue experiment, OVCAR5 Pt-R cells were plated at 50,000 cells/well. The following day, the media was changed to media containing 1% FBS and 24 hours later 50 μg/mL cholesterol was added. 48 hours later, cells were incubated with BODIPY 581/591 C11 (2.5 μM) at 37°C for 1 hr and washed with PBS. The mean fluorescence intensity (MFI) was measured by FACS (LSR Fortessa, BD, Franklin Lake, NJ) at a minimum of 10,000 events per sample. FITC (520 nm; oxidized BODIPY) and PE (580 nm; reduced BODIPY) emissions were recorded. The data were displayed as histograms and mean fluorescence intensity of FITC and the ratio of oxidized to reduced C11 was calculated. Data were analyzed using FlowJo software.
